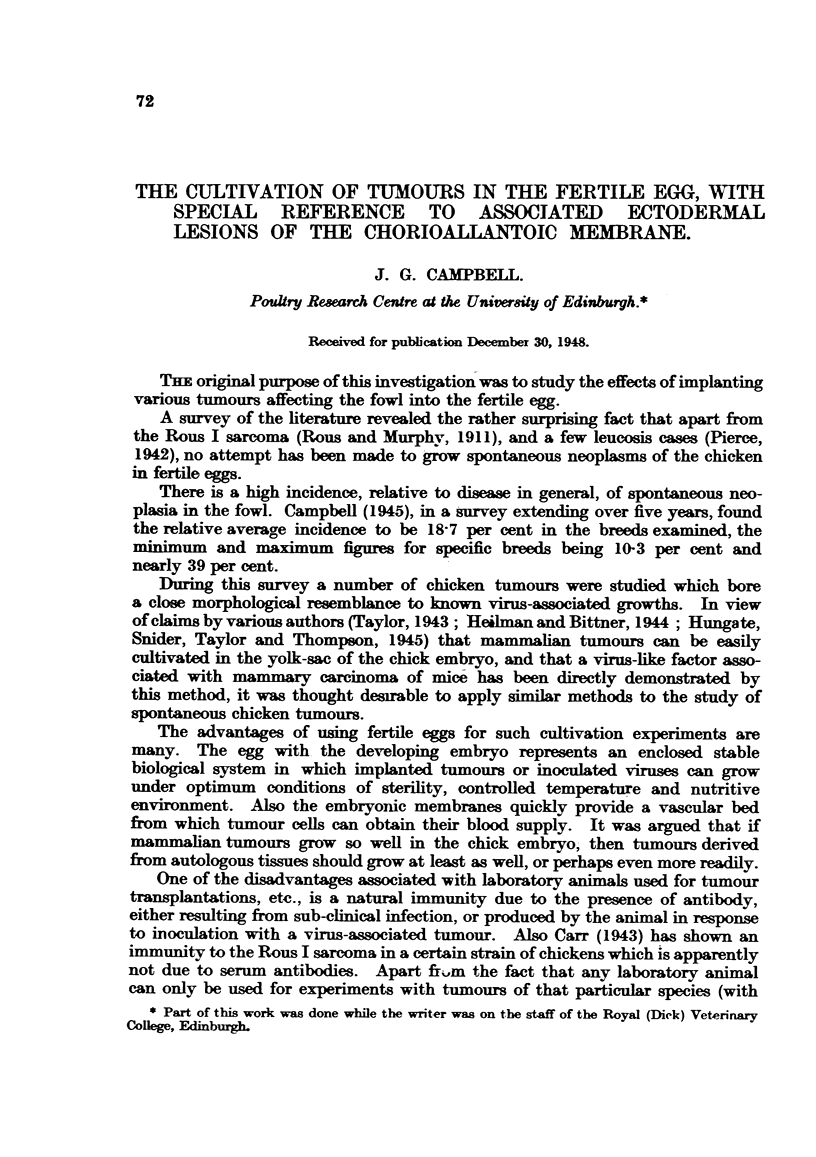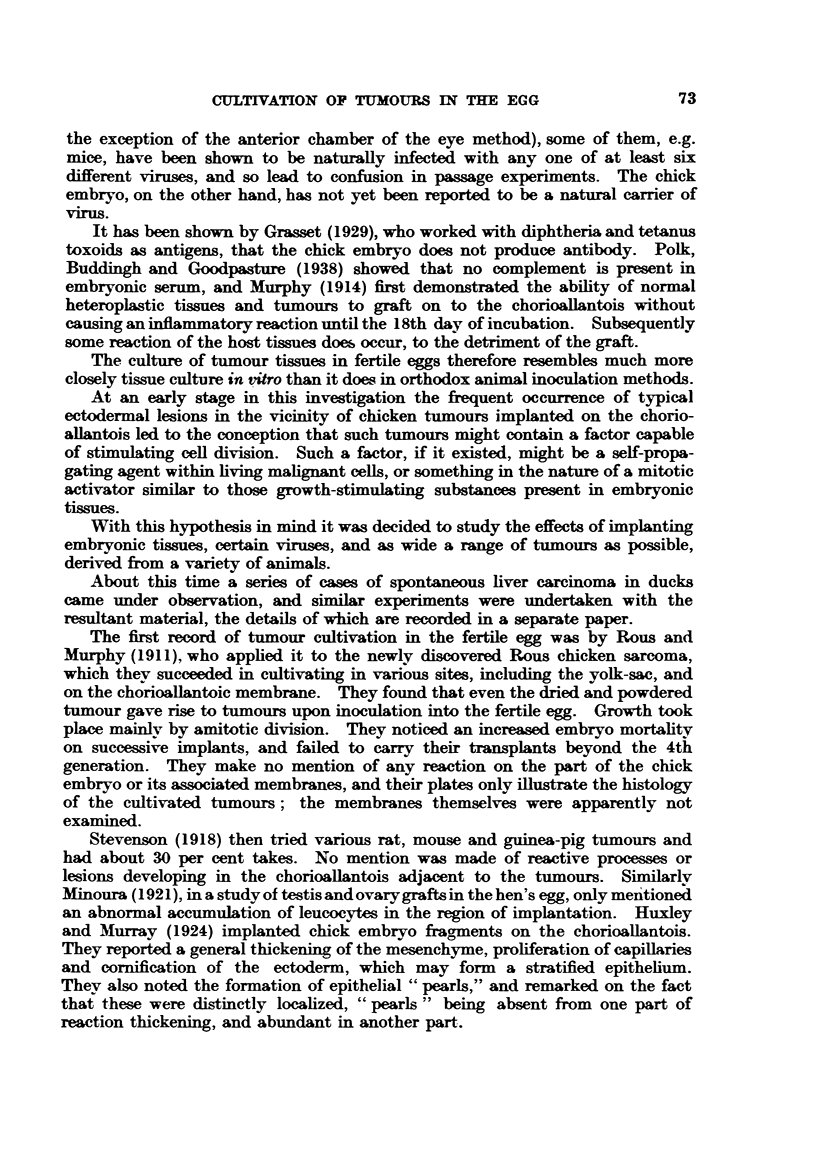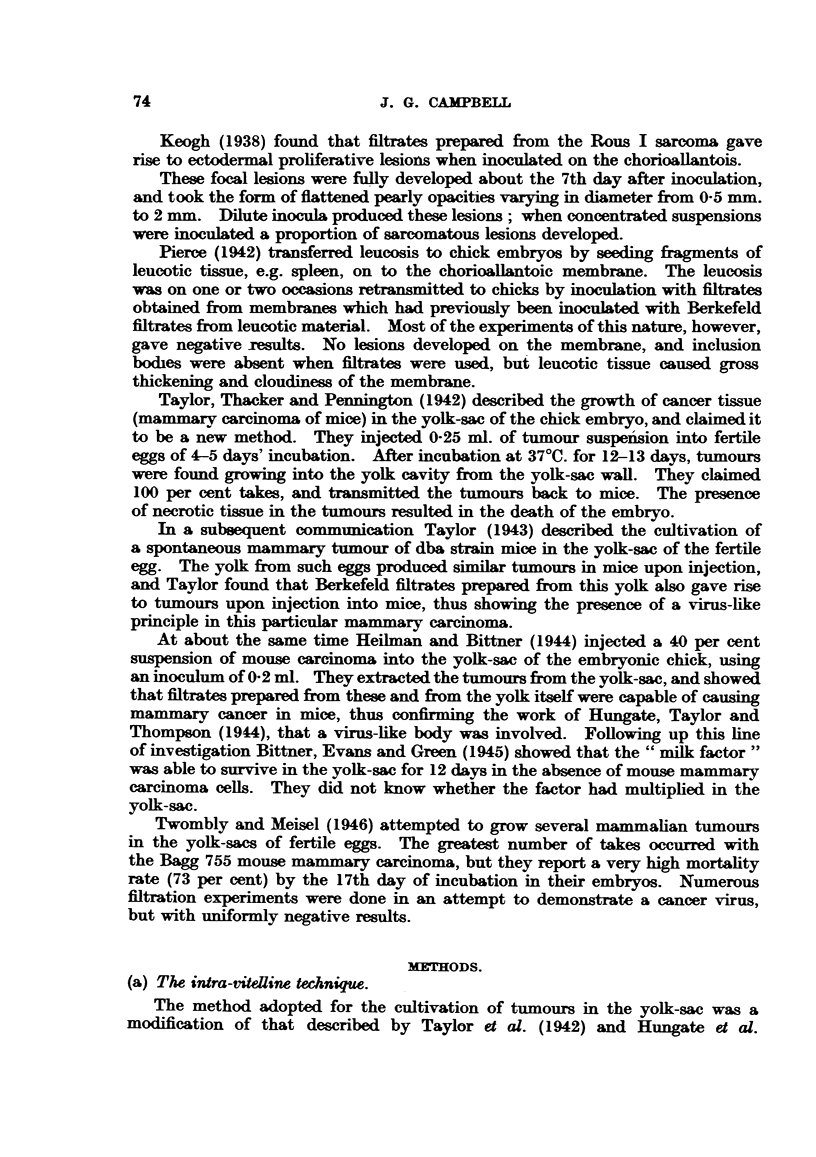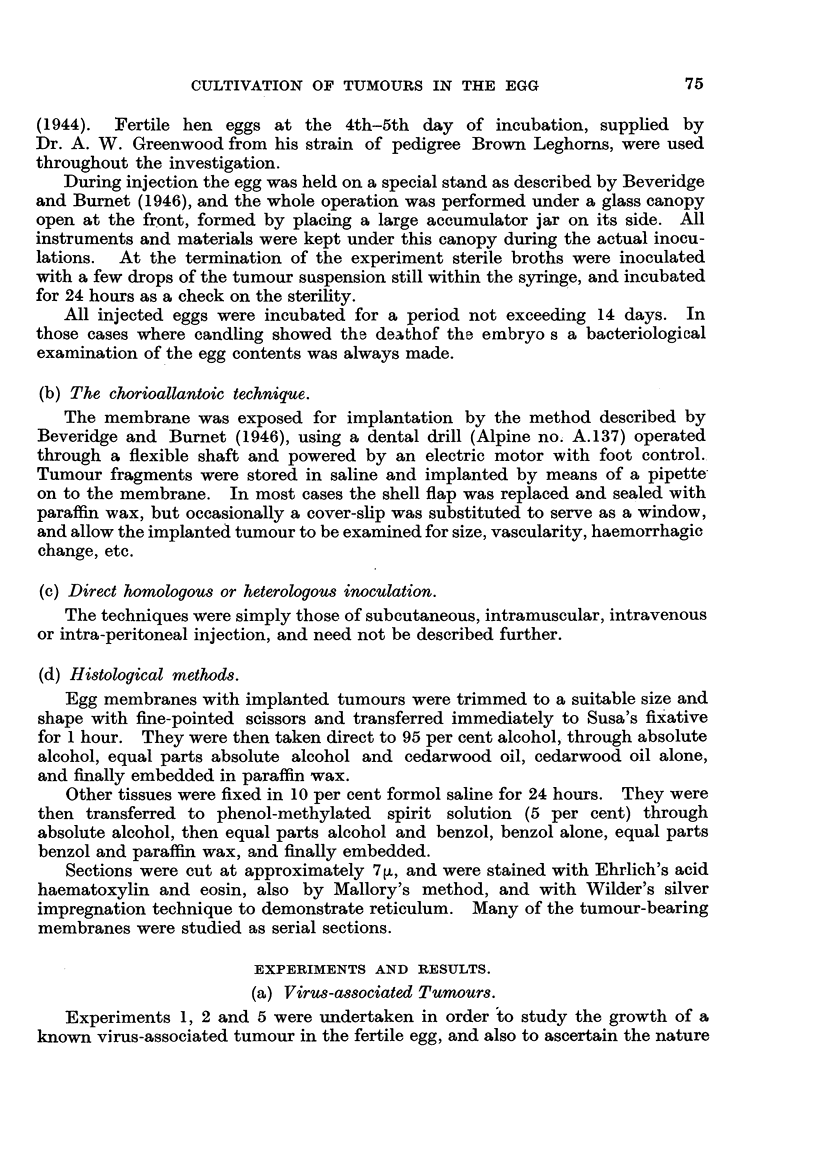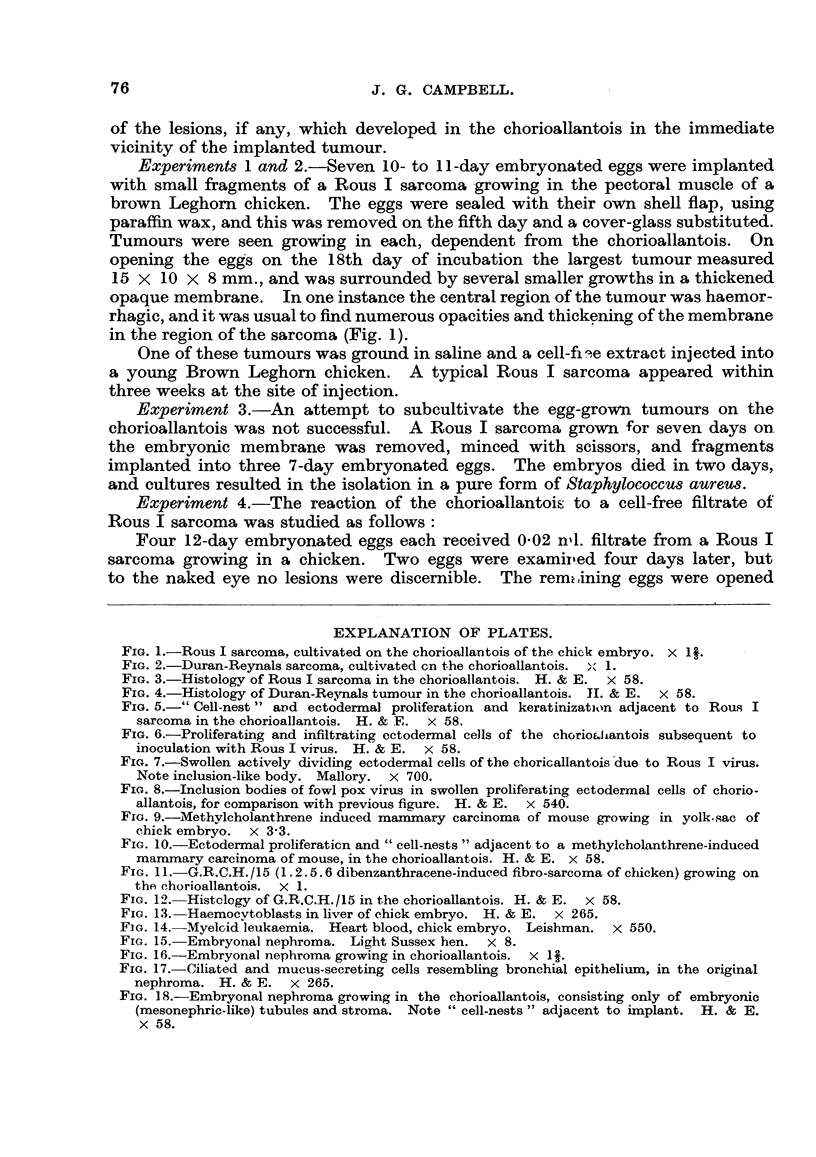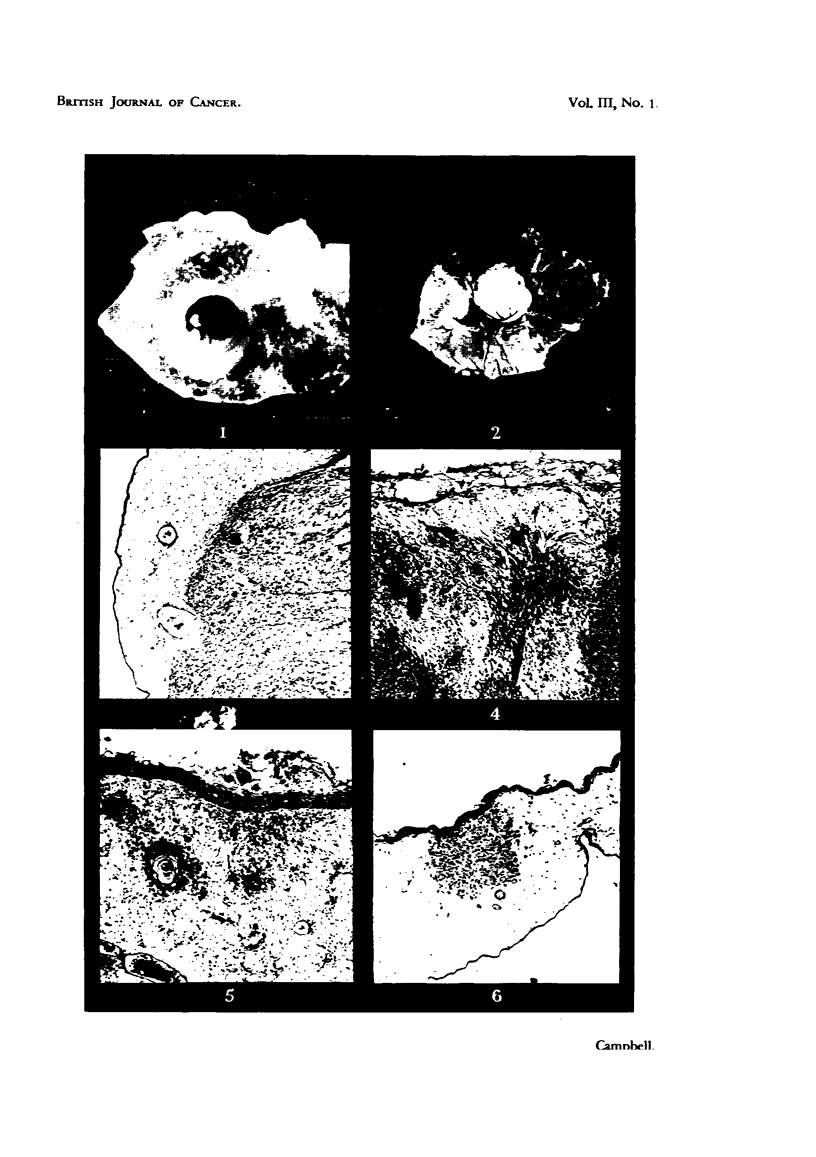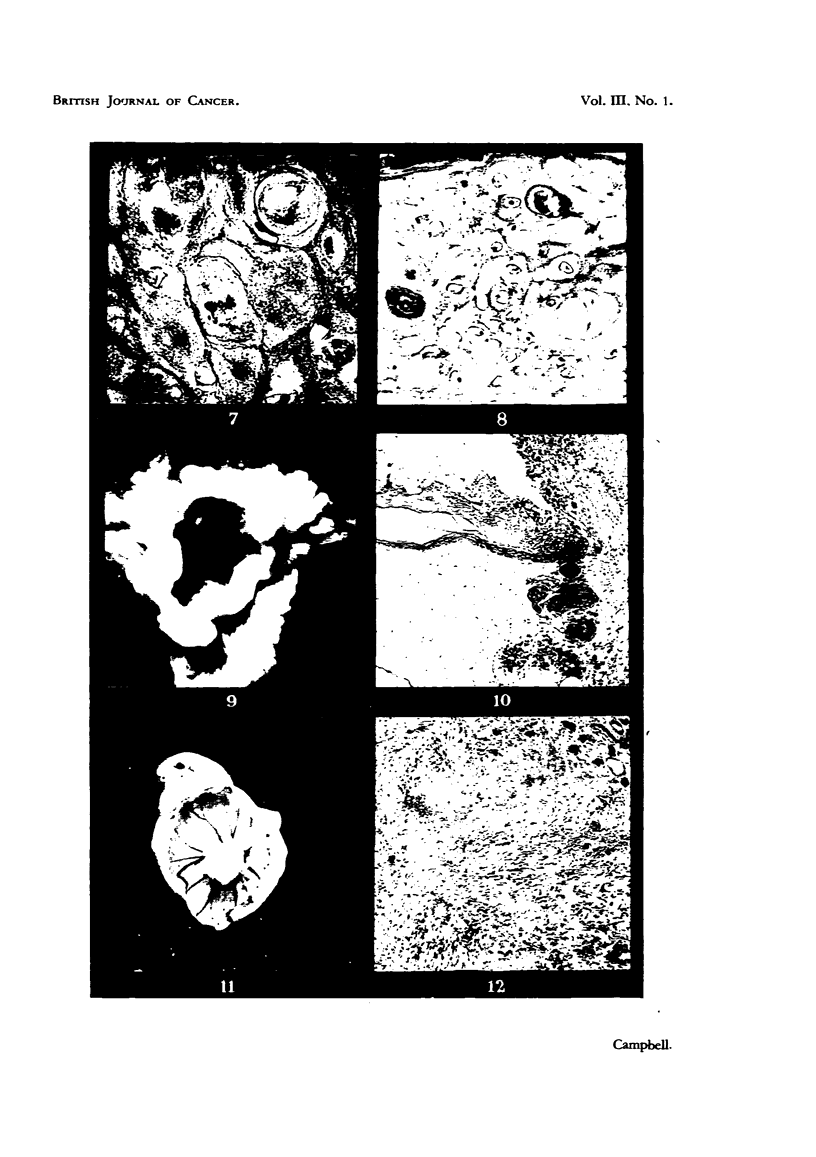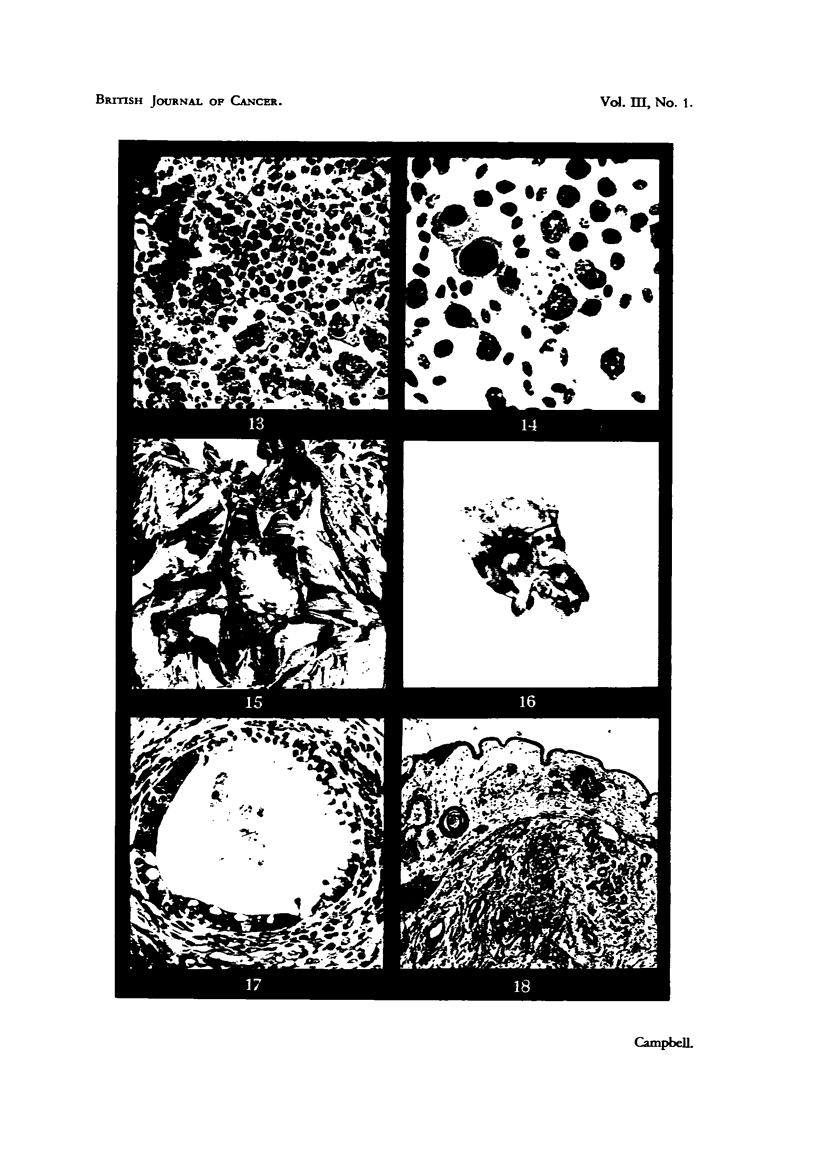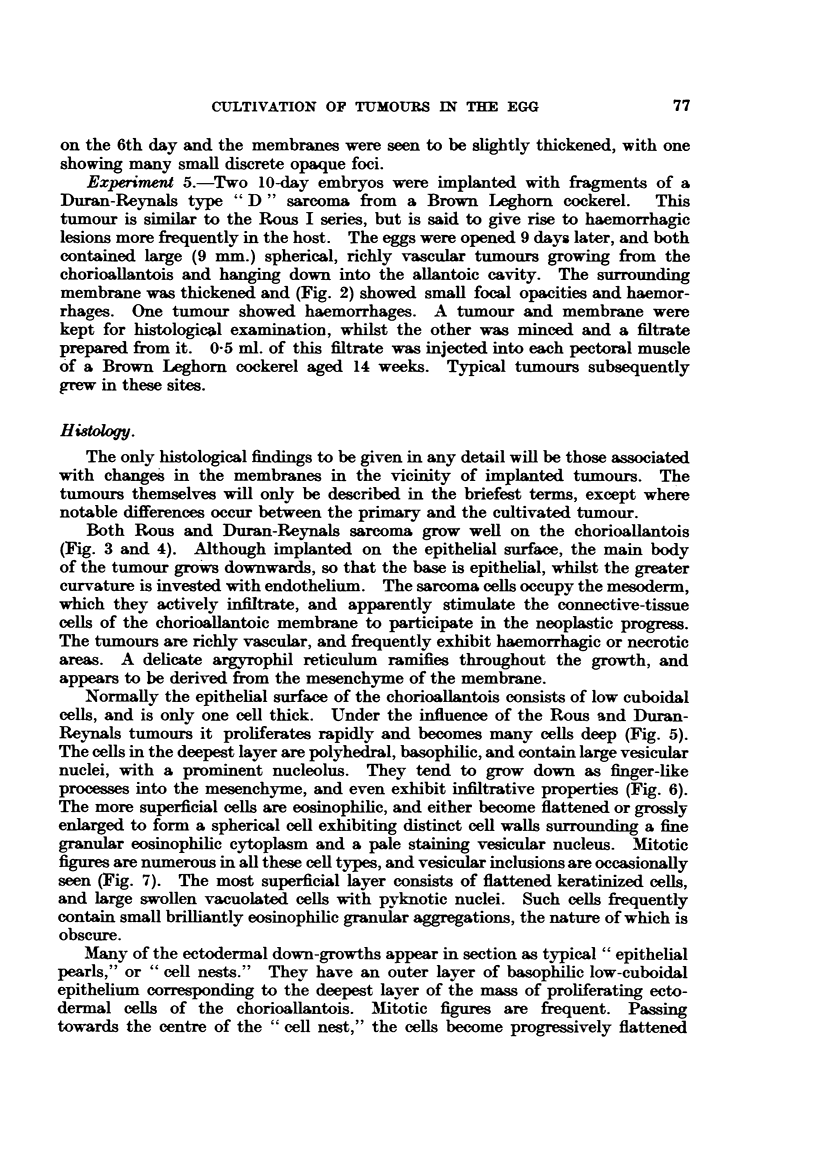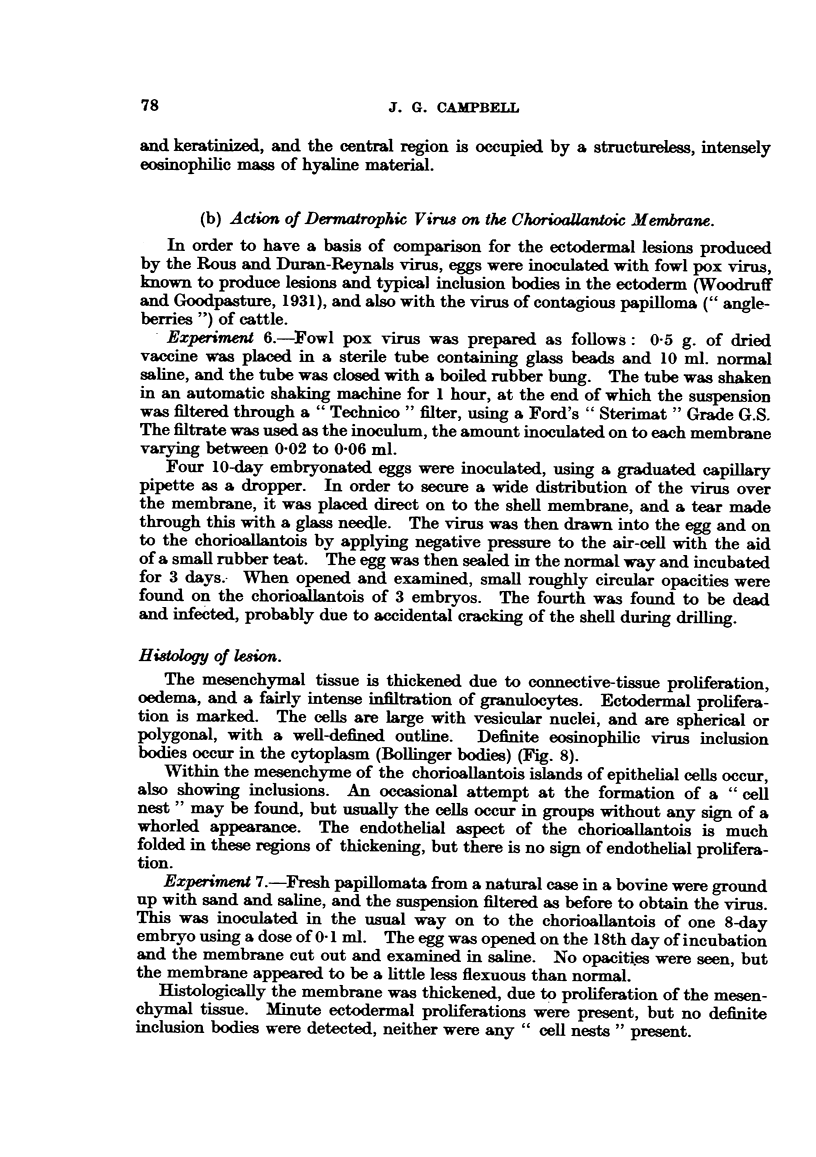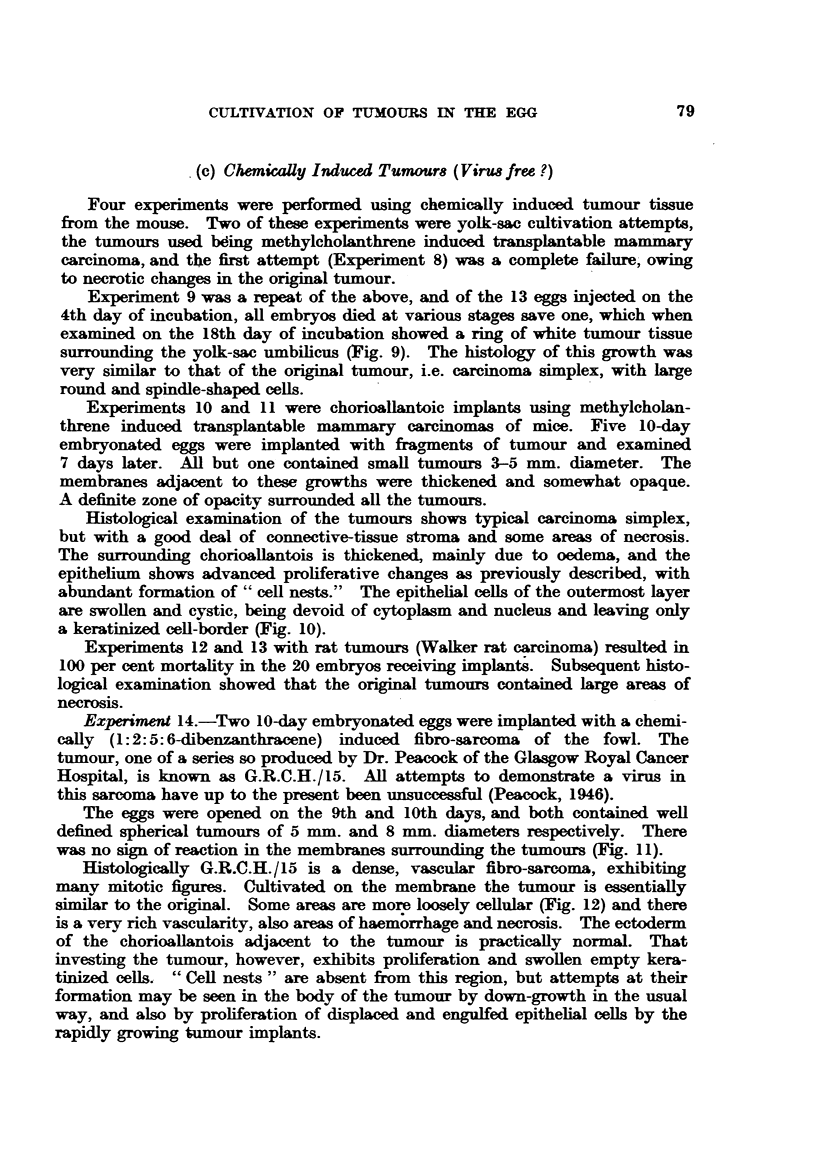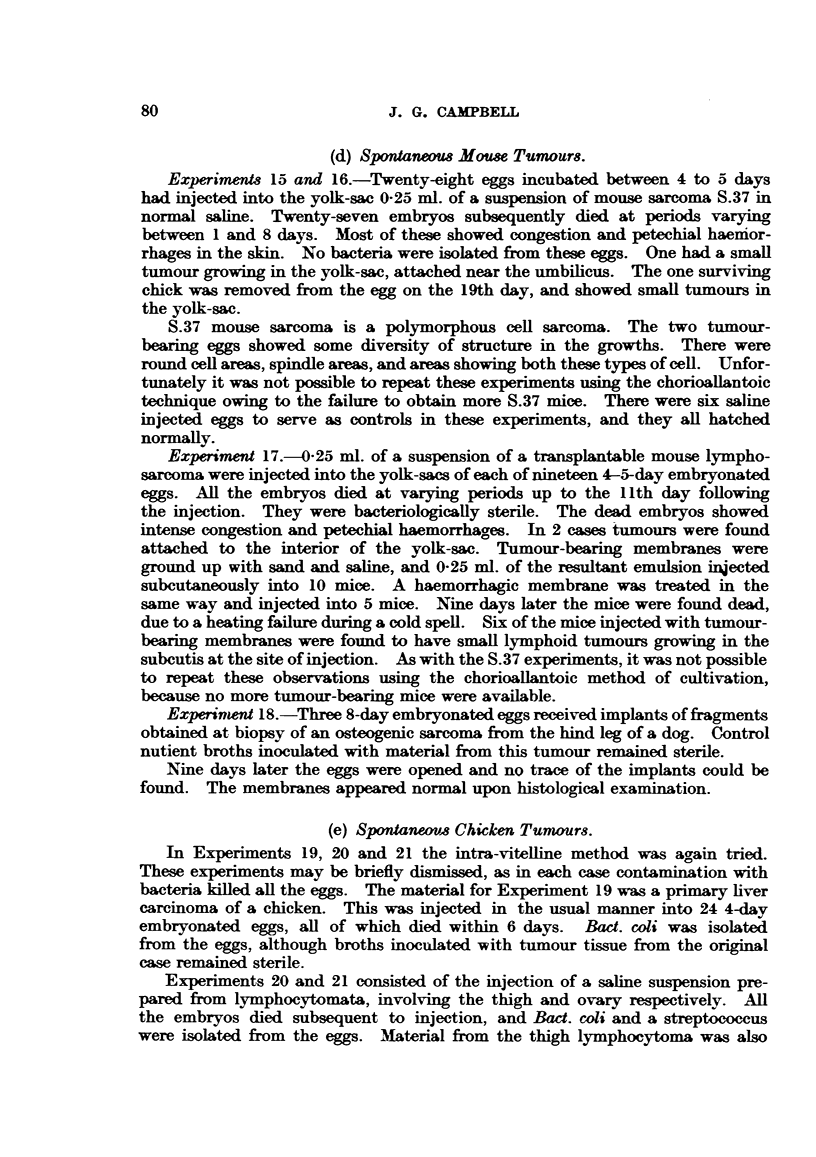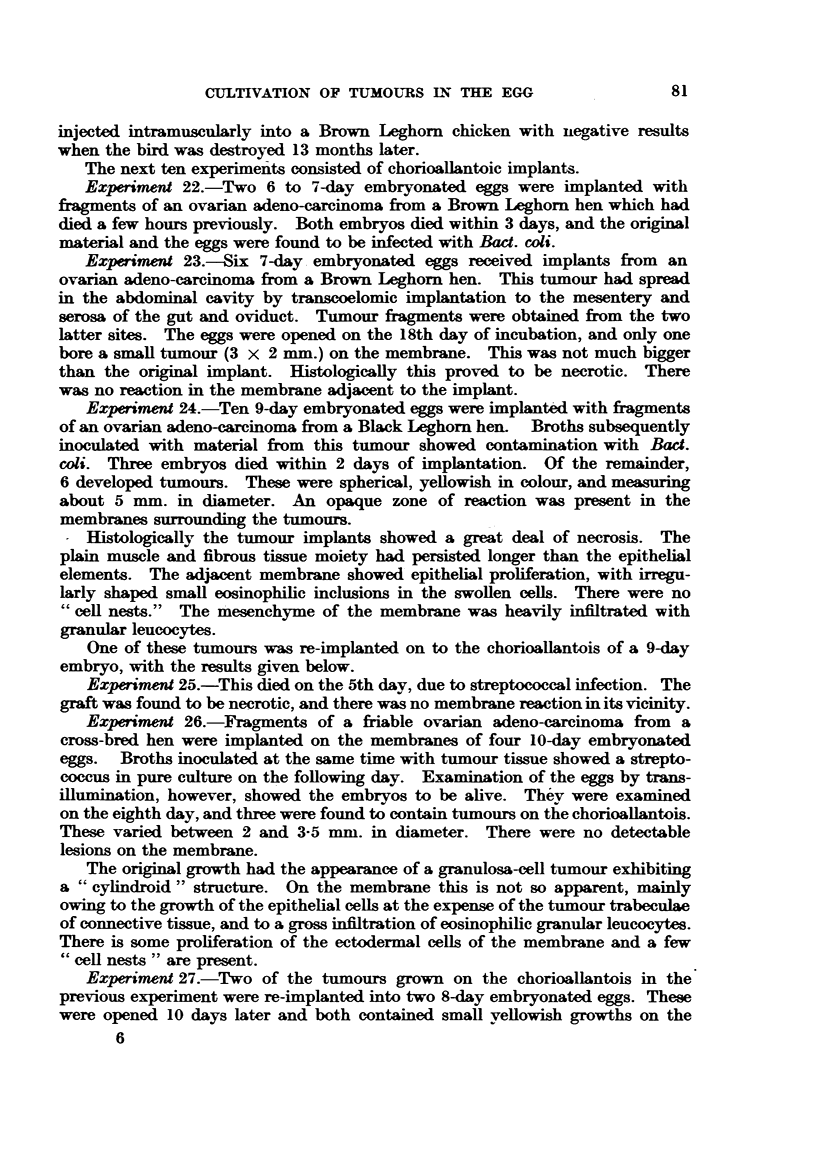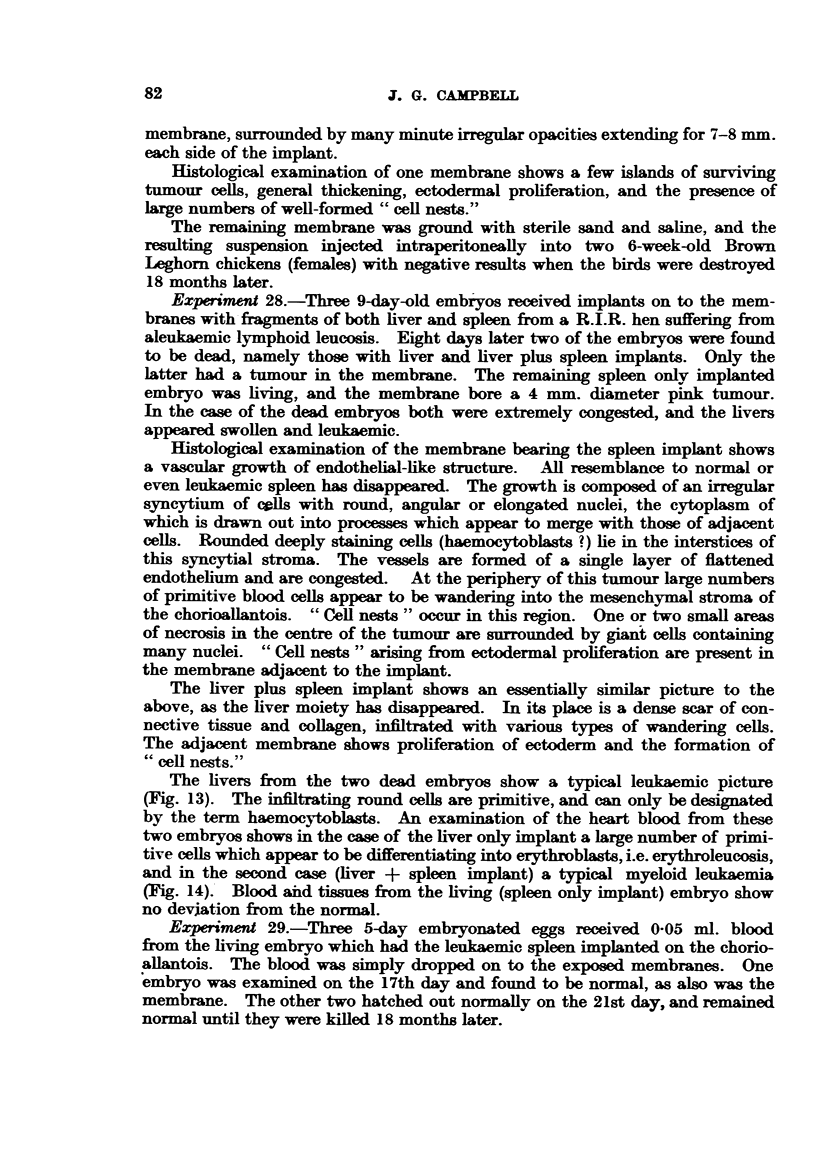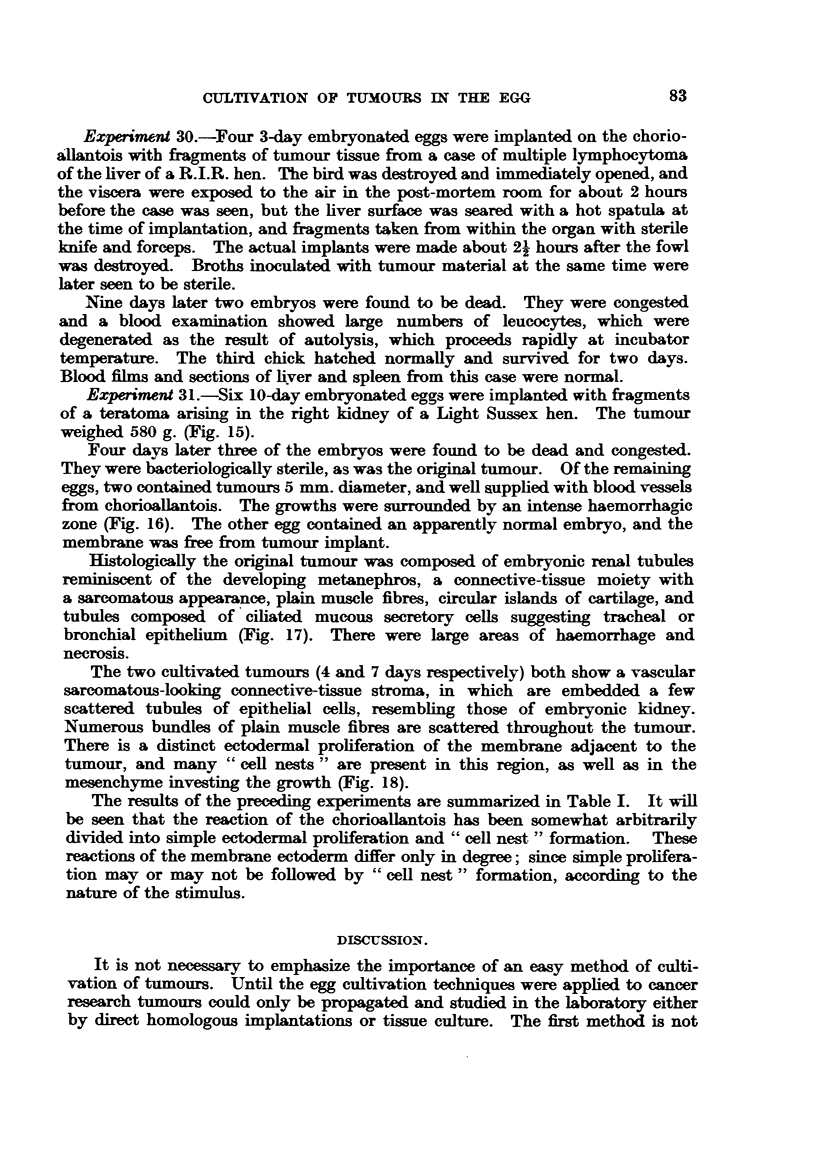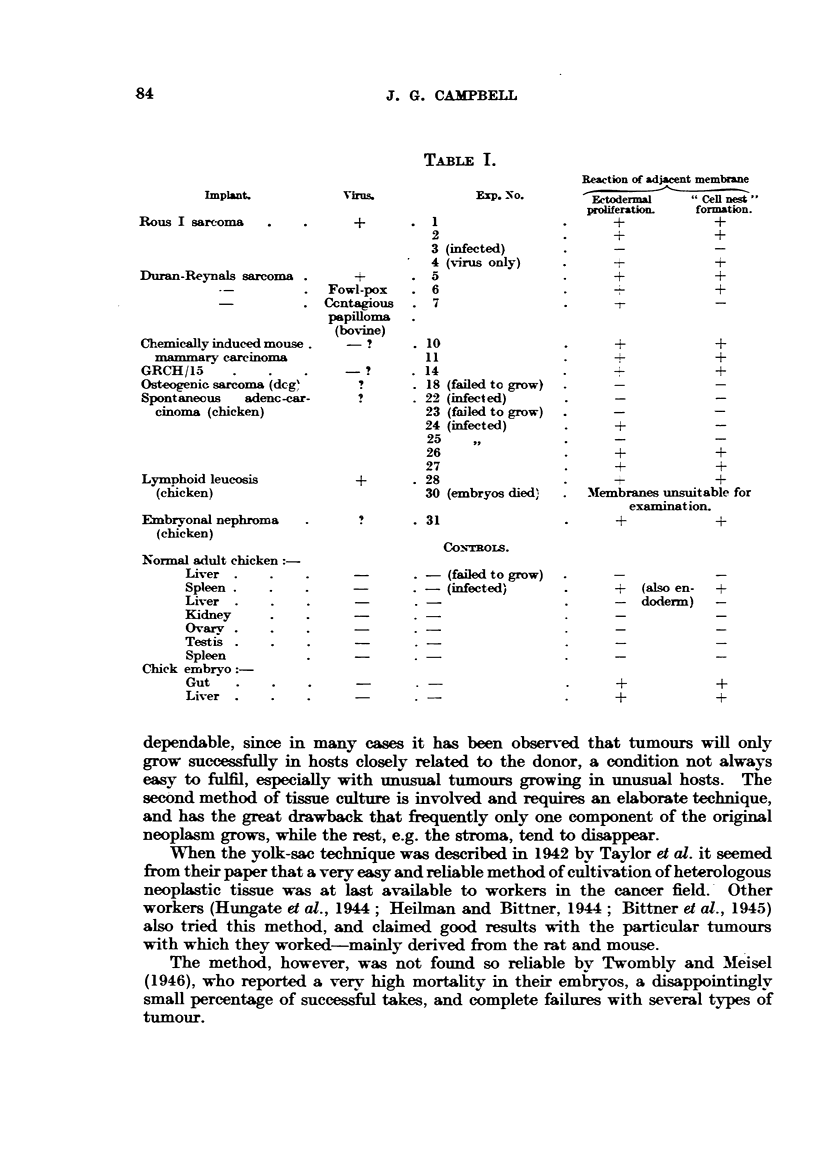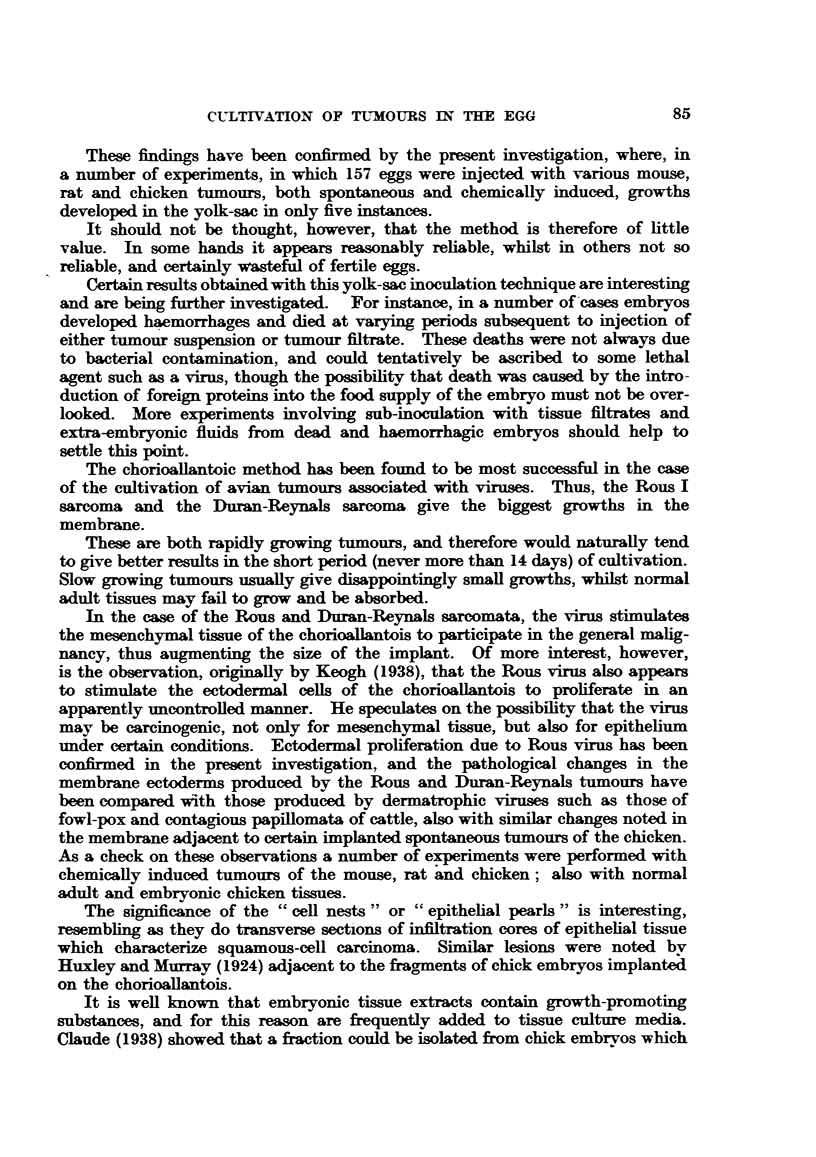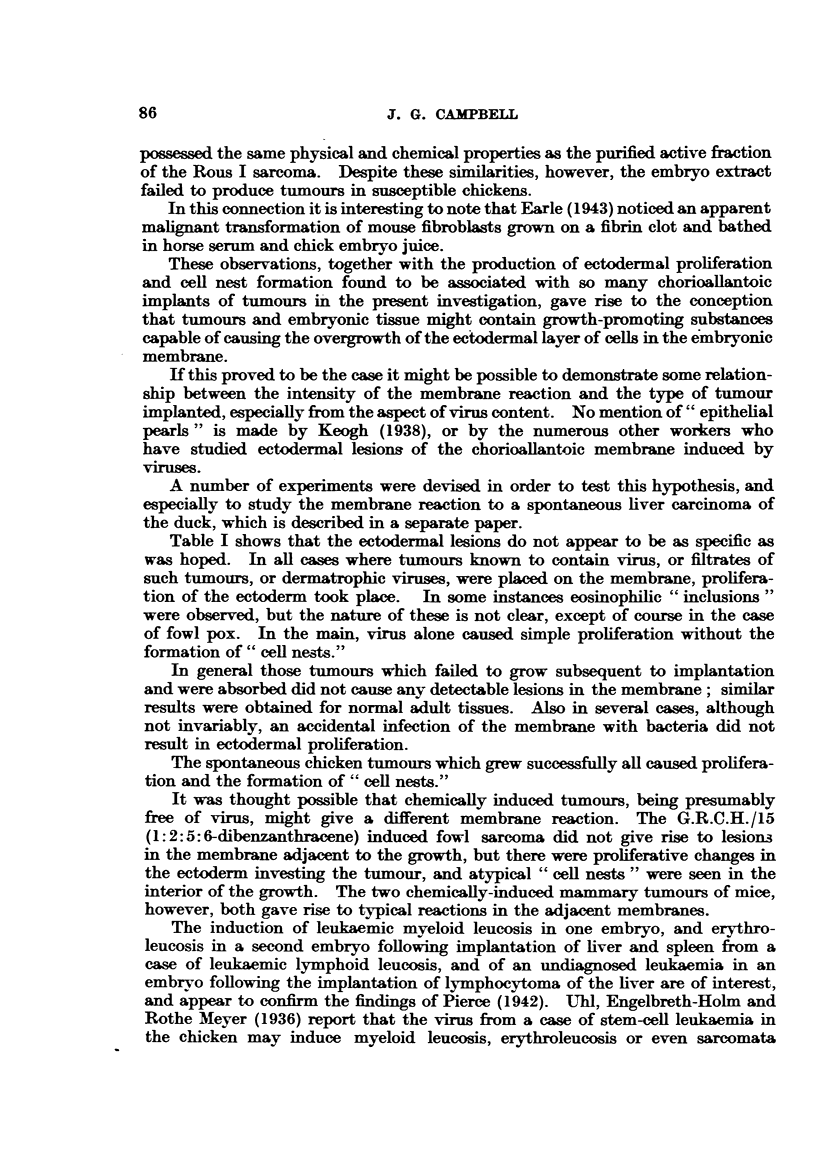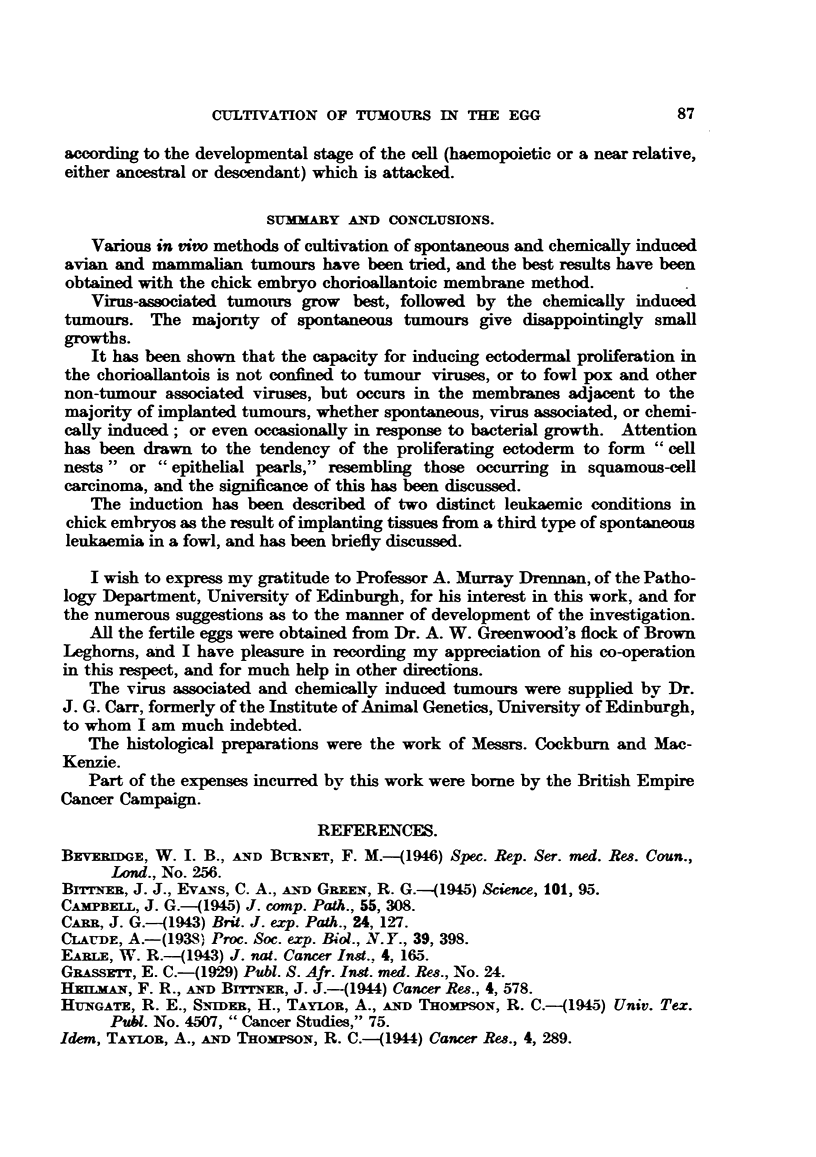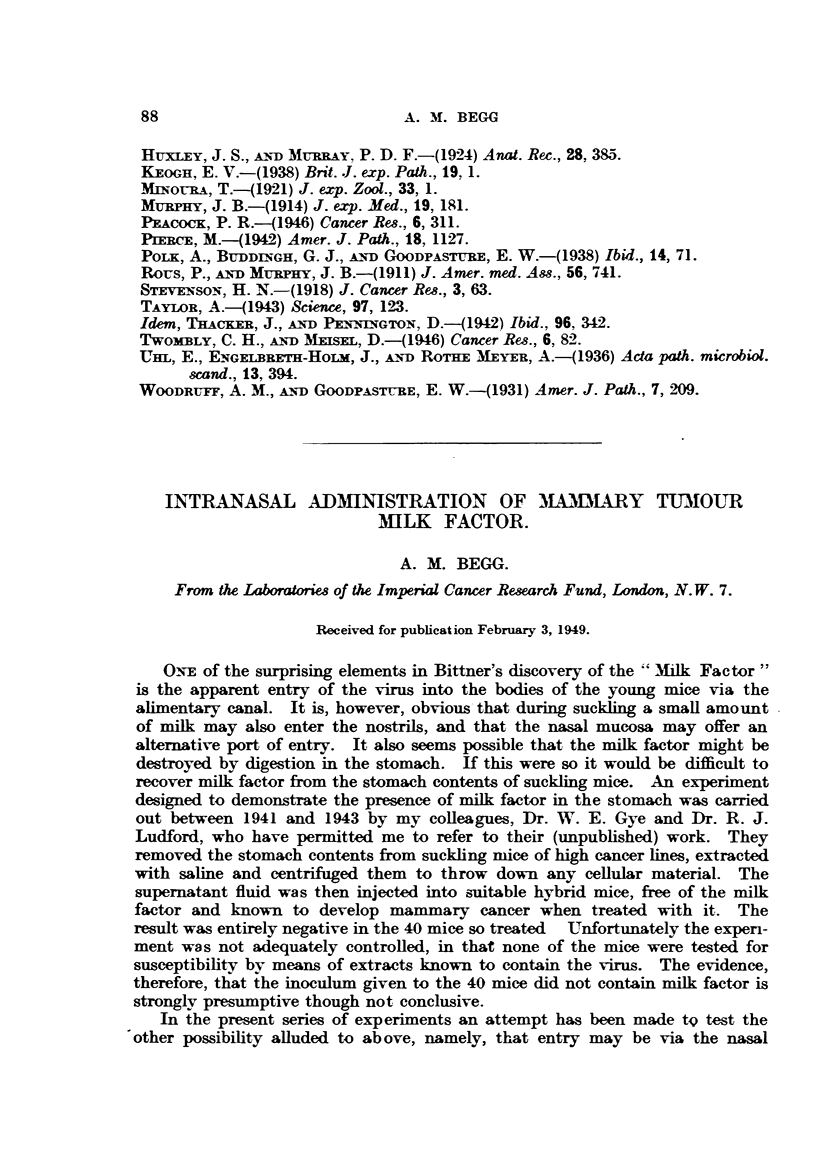# The Cultivation of Tumours in the Fertile Egg, with Special Reference to Associated Ectodermal Lesions of the Chorioallantoic Membrane

**DOI:** 10.1038/bjc.1949.8

**Published:** 1949-03

**Authors:** J. G. Campbell

## Abstract

**Images:**


### THE CULTIVATION OF TUMOURS IN THE FERTILE EGG, WITH SPECIAL REFERENCE TO ASSOCIATED ECTODERMAL LESIONS OF THE CHORIOALLANTOIC MEMBRANE.

J. G. CAMPBELL.

*Poultry Research Centre at the University of Edinburgh.**

Received for publication December 30, 1948.

THE original purpose of this investigation was to study the effects of implanting various tumours affecting the fowl into the fertile egg.

A survey of the literature revealed the rather surprising fact that apart from the Rous I sarcoma (Rous and Murphy, 1911), and a few leucosis cases (Pierce, 1942), no attempt has been made to grow spontaneous neoplasms of the chicken in fertile eggs.

There is a high incidence, relative to disease in general, of spontaneous neoplasia in the fowl. Campbell (1945), in a survey extending over five years, found the relative average incidence to be 18·7 per cent in the breeds examined, the minimum and maximum figures for specific breeds being 10·3 per cent and nearly 39 per cent.

During this survey a number of chicken tumours were studied which bore a close morphological resemblance to known virus-associated growths. In view of claims by various authors (Taylor, 1943 ; Heilman and Bittner, 1944 ; Hungate, Snider, Taylor and Thompson, 1945) that mammalian tumours can be easily cultivated in the yolk-sac of the chick embryo, and that a virus-like factor associated with mammary carcinoma of mice has been directly demonstrated by this method, it was thought desirable to apply similar methods to the study of spontaneous chicken tumours.

The advantages of using fertile eggs for such cultivation experiments are many. The egg with the developing embryo represents an enclosed stable biological system in which implanted tumours or inoculated viruses can grow under optimum conditions of sterility, controlled temperature and nutritive environment. Also the embryonic membranes quickly provide a vascular bed from which tumour cells can obtain their blood supply. It was argued that if mammalian tumours grow so well in the chick embryo, then tumours derived from autologous tissues should grow at least as well, or perhaps even more readily.

One of the disadvantages associated with laboratory animals used for tumour transplantations, etc., is a natural immunity due to the presence of antibody, either resulting from sub-clinical infection, or produced by the animal in response to inoculation with a virus-associated tumour. Also Carr (1943) has shown an immunity to the Rous I sarcoma in a certain strain of chickens which is apparently not due to serum antibodies. Apart from the fact that any laboratory animal can only be used for experiments with tumours of that particular species (with

* Part of this work was done while the writer was on the staff of the Royal (Dick) Veterinary College, Edinburgh.

the exception of the anterior chamber of the eye method), some of them, e.g. mice, have been shown to be naturally infected with any one of at least six different viruses, and so lead to confusion in passage experiments. The chick embryo, on the other hand, has not yet been reported to be a natural carrier of virus.

It has been shown by Grasset (1929), who worked with diphtheria and tetanus toxoids as antigens, that the chick embryo does not produce antibody. Polk, Buddingh and Goodpasture (1938) showed that no complement is present in embryonic serum, and Murphy (1914) first demonstrated the ability of normal heteroplastic tissues and tumours to graft on to the chorioallantois without causing an inflammatory reaction until the 18th day of incubation. Subsequently some reaction of the host tissues does occur, to the detriment of the graft.

The culture of tumour tissues in fertile eggs therefore resembles much more closely tissue culture *in vitro* than it does in orthodox animal inoculation methods.

At an early stage in this investigation the frequent occurrence of typical ectodermal lesions in the vicinity of chicken tumours implanted on the chorio-allantois led to the conception that such tumours might contain a factor capable of stimulating cell division. Such a factor, if it existed, might be a self-propagating agent within living malignant cells, or something in the nature of a mitotic activator similar to those growth-stimulating substances present in embryonic tissues.

With this hypothesis in mind it was decided to study the effects of implanting embryonic tissues, certain viruses, and as wide a range of tumours as possible, derived from a variety of animals.

About this time a series of cases of spontaneous liver carcinoma in ducks came under observation, and similar experiments were undertaken with the resultant material, the details of which are recorded in a separate paper.

The first record of tumour cultivation in the fertile egg was by Rous and Murphy (1911), who applied it to the newly discovered Rous chicken sarcoma, which they succeeded in cultivating in various sites, including the yolk-sac, and on the chorioallantoic membrane. They found that even the dried and powdered tumour gave rise to tumours upon inoculation into the fertile egg. Growth took place mainly by amitotic division. They noticed an increased embryo mortality on successive implants, and failed to carry their transplants beyond the 4th generation. They make no mention of any reaction on the part of the chick embryo or its associated membranes, and their plates only illustrate the histology of the cultivated tumours; the membranes themselves were apparently not examined.

Stevenson (1918) then tried various rat, mouse and guinea-pig tumours and had about 30 per cent takes. No mention was made of reactive processes or lesions developing in the chorioallantois adjacent to the tumours. Similarly Minoura (1921), in a study of testis and ovary grafts in the hen's egg, only mentioned an abnormal accumulation of leucocytes in the region of implantation. Huxley and Murray (1924) implanted chick embryo fragments on the chorioallantois. They reported a general thickening of the mesenchyme, proliferation of capillaries and cornification of the ectoderm, which may form a stratified epithelium. They also noted the formation of epithelial "pearls," and remarked on the fact that these were distinctly localized, "pearls" being absent from one part of reaction thickening, and abundant in another part.

Keogh (1938) found that filtrates prepared from the Rous I sarcoma gave rise to ectodermal proliferative lesions when inoculated on the chorioallantois.

These focal lesions were fully developed about the 7th day after inoculation, and took the form of flattened pearly opacities varying in diameter from 0·5 mm. to 2 mm. Dilute inocula produced these lesions ; when concentrated suspensions were inoculated a proportion of sarcomatous lesions developed.

Pierce (1942) transferred leucosis to chick embryos by seeding fragments of leucotic tissue, e.g. spleen, on to the chorioallantoic membrane. The leucosis was on one or two occasions retransmitted to chicks by inoculation with filtrates obtained from membranes which had previously been inoculated with Berkefeld filtrates from leucotic material. Most of the experiments of this nature, however, gave negative results. No lesions developed on the membrane, and inclusion bodies were absent when filtrates were used, but leucotic tissue caused gross thickening and cloudiness of the membrane.

Taylor, Thacker and Pennington (1942) described the growth of cancer tissue (mammary carcinoma of mice) in the yolk-sac of the chick embryo, and claimed it to be a new method. They injected 0·25 ml. of tumour suspension into fertile eggs of 4–5 days' incubation. After incubation at 37°C. for 12–13 days, tumours were found growing into the yolk cavity from the yolk-sac wall. They claimed 100 per cent takes, and transmitted the tumours back to mice. The presence of necrotic tissue in the tumours resulted in the death of the embryo.

In a subsequent communication Taylor (1943) described the cultivation of a spontaneous mammary tumour of dba strain mice in the yolk-sac of the fertile egg. The yolk from such eggs produced similar tumours in mice upon injection, and Taylor found that Berkefeld filtrates prepared from this yolk also gave rise to tumours upon injection into mice, thus showing the presence of a virus-like principle in this particular mammary carcinoma.

At about the same time Heilman and Bittner (1944) injected a 40 per cent suspension of mouse carcinoma into the yolk-sac of the embryonic chick, using an inoculum of 0·2 ml. They extracted the tumours from the yolk-sac, and showed that filtrates prepared from these and from the yolk itself were capable of causing mammary cancer in mice, thus confirming the work of Hungate, Taylor and Thompson (1944), that a virus-like body was involved. Following up this line of investigation Bittner, Evans and Green (1945) showed that the " milk factor " was able to survive in the yolk-sac for 12 days in the absence of mouse mammary carcinoma cells. They did not know whether the factor had multiplied in the yolk-sac.

Twombly and Meisel (1946) attempted to grow several mammalian tumours in the yolk-sacs of fertile eggs. The greatest number of takes occurred with the Bagg 755 mouse mammary carcinoma, but they report a very high mortality rate (73 per cent) by the 17th day of incubation in their embryos. Numerous filtration experiments were done in an attempt to demonstrate a cancer virus, but with uniformly negative results.

### METHODS.

### (a) *The intra-vitelline technique.*

The method adopted for the cultivation of tumours in the yolk-sac was a modification of that described by Taylor *et al.* (1942) and Hungate *et al.*

(1944). Fertile hen eggs at the 4th–5th day of incubation, supplied by Dr. A. W. Greenwood from his strain of pedigree Brown Leghorns, were used throughout the investigation.

During injection the egg was held on a special stand as described by Beveridge and Burnet (1946), and the whole operation was performed under a glass canopy open at the front, formed by placing a large accumulator jar on its side. All instruments and materials were kept under this canopy during the actual inoculations. At the termination of the experiment sterile broths were inoculated with a few drops of the tumour suspension still within the syringe, and incubated for 24 hours as a check on the sterility.

All injected eggs were incubated for a period not exceeding 14 days. In those cases where candling showed the deathof the embryo s a bacteriological examination of the egg contents was always made.

(b) *The chorioallantoic technique.*

The membrane was exposed for implantation by the method described by Beveridge and Burnet (1946), using a dental drill (Alpine no. A.137) operated through a flexible shaft and powered by an electric motor with foot control. Tumour fragments were stored in saline and implanted by means of a pipette on to the membrane. In most cases the shell flap was replaced and sealed with paraffin wax, but occasionally a cover-slip was substituted to serve as a window, and allow the implanted tumour to be examined for size, vascularity, haemorrhagic change, etc.

(c) *Direct homologous or heterologous inoculation.*

The techniques were simply those of subcutaneous, intramuscular, intravenous or intra-peritoneal injection, and need not be described further.

(d) *Histological methods.*

Egg membranes with implanted tumours were trimmed to a suitable size and shape with fine-pointed scissors and transferred immediately to Susa's fixative for 1 hour. They were then taken direct to 95 per cent alcohol, through absolute alcohol, equal parts absolute alcohol and cedarwood oil, cedarwood oil alone, and finally embedded in paraffin wax.

Other tissues were fixed in 10 per cent formol saline for 24 hours. They were then transferred to phenol-methylated spirit solution (5 per cent) through absolute alcohol, then equal parts alcohol and benzol, benzol alone, equal parts benzol and paraffin wax, and finally embedded.

Sections were cut at approximately 7$\mu$, and were stained with Ehrlich's acid haematoxylin and eosin, also by Mallory's method, and with Wilder's silver impregnation technique to demonstrate reticulum. Many of the tumour-bearing membranes were studied as serial sections.

### EXPERIMENTS AND RESULTS.

### (a) *Virus-associated Tumours.*

Experiments 1, 2 and 5 were undertaken in order to study the growth of a known virus-associated tumour in the fertile egg, and also to ascertain the nature of the lesions, if any, which developed in the chorioallantois in the immediate vicinity of the implanted tumour.

*Experiments* 1 *and* 2.—Seven 10- to 11-day embryonated eggs were implanted with small fragments of a Rous I sarcoma growing in the pectoral muscle of a brown Leghorn chicken. The eggs were sealed with their own shell flap, using paraffin wax, and this was removed on the fifth day and a cover-glass substituted. Tumours were seen growing in each, dependent from the chorioallantois. On opening the eggs on the 18th day of incubation the largest tumour measured 15 × 10 × 8 mm., and was surrounded by several smaller growths in a thickened opaque membrane. In one instance the central region of the tumour was haemorrhagic, and it was usual to find numerous opacities and thickening of the membrane in the region of the sarcoma (Fig. 1).

One of these tumours was ground in saline and a cell-free extract injected into a young Brown Leghorn chicken. A typical Rous I sarcoma appeared within three weeks at the site of injection.

*Experiment* 3.—An attempt to subcultivate the egg-grown tumours on the chorioallantois was not successful. A Rous I sarcoma grown for seven days on the embryonic membrane was removed, minced with scissors, and fragments implanted into three 7-day embryonated eggs. The embryos died in two days, and cultures resulted in the isolation in a pure form of *Staphylococcus aureus.*

*Experiment* 4.—The reaction of the chorioallantois to a cell-free filtrate of Rous I sarcoma was studied as follows :

Four 12-day embryonated eggs each received 0·02 ml. filtrate from a Rous I sarcoma growing in a chicken. Two eggs were examined four days later, but to the naked eye no lesions were discernible. The remaining eggs were opened

EXPLANATION OF PLATES.

FIG. 1.—Rous I sarcoma, cultivated on the chorioallantois of the chick embryo. × 1⅗.
FIG. 2.—Duran-Reynals sarcoma, cultivated on the chorioallantois. × 1.
FIG. 3.—Histology of Rous I sarcoma in the chorioallantois. H. & E. × 58.
FIG. 4.—Histology of Duran-Reynals tumour in the chorioallantois. H. & E. × 58.
FIG. 5.—" Cell-nest " and ectodermal proliferation and keratinization adjacent to Rous I sarcoma in the chorioallantois. H. & E. × 58.
FIG. 6.—Proliferating and infiltrating ectodermal cells of the chorioallantois subsequent to inoculation with Rous I virus. H. & E. × 58.
FIG. 7.—Swollen actively dividing ectodermal cells of the chorioallantois due to Rous I virus. Note inclusion-like body. Mallory. × 700.
FIG. 8.—Inclusion bodies of fowl pox virus in swollen proliferating ectodermal cells of chorioallantois, for comparison with previous figure. H. & E. × 540.
FIG. 9.—Methylcholanthrene induced mammary carcinoma of mouse growing in yolk-sac of chick embryo. × 3·3.
FIG. 10.—Ectodermal proliferation and " cell-nests " adjacent to a methylcholanthrene-induced mammary carcinoma of mouse, in the chorioallantois. H. & E. × 58.
FIG. 11.—G.R.C.H./15 (1.2.5.6 dibenzanthracene-induced fibro-sarcoma of chicken) growing on the chorioallantois. × 1.
FIG. 12.—Histology of G.R.C.H./15 in the chorioallantois. H. & E. × 58.
FIG. 13.—Haemocytoblasts in liver of chick embryo. H. & E. × 265.
FIG. 14.—Myeloid leukaemia. Heart blood, chick embryo. Leishman. × 550.
FIG. 15.—Embryonal nephroma. Light Sussex hen. × 8.
FIG. 16.—Embryonal nephroma growing in chorioallantois. × 1⅗.
FIG. 17.—Ciliated and mucus-secreting cells resembling bronchial epithelium, in the original nephroma. H. & E. × 265.
FIG. 18.—Embryonal nephroma growing in the chorioallantois, consisting only of embryonic (mesonephric-like) tubules and stroma. Note " cell-nests " adjacent to implant. H. & E. × 58.

1

2

3

4

5

6

7

8

9

10

11

12

13
14
15
16
17
18

on the 6th day and the membranes were seen to be slightly thickened, with one showing many small discrete opaque foci.

*Experiment* 5.—Two 10-day embryos were implanted with fragments of a Duran-Reynals type " D " sarcoma from a Brown Leghorn cockerel. This tumour is similar to the Rous I series, but is said to give rise to haemorrhagic lesions more frequently in the host. The eggs were opened 9 days later, and both contained large (9 mm.) spherical, richly vascular tumours growing from the chorioallantois and hanging down into the allantoic cavity. The surrounding membrane was thickened and (Fig. 2) showed small focal opacities and haemorrhages. One tumour showed haemorrhages. A tumour and membrane were kept for histological examination, whilst the other was minced and a filtrate prepared from it. 0·5 ml. of this filtrate was injected into each pectoral muscle of a Brown Leghorn cockerel aged 14 weeks. Typical tumours subsequently grew in these sites.

*Histology.*

The only histological findings to be given in any detail will be those associated with changes in the membranes in the vicinity of implanted tumours. The tumours themselves will only be described in the briefest terms, except where notable differences occur between the primary and the cultivated tumour.

Both Rous and Duran-Reynals sarcoma grow well on the chorioallantois (Fig. 3 and 4). Although implanted on the epithelial surface, the main body of the tumour grows downwards, so that the base is epithelial, whilst the greater curvature is invested with endothelium. The sarcoma cells occupy the mesoderm, which they actively infiltrate, and apparently stimulate the connective-tissue cells of the chorioallantoic membrane to participate in the neoplastic progress. The tumours are richly vascular, and frequently exhibit haemorrhagic or necrotic areas. A delicate argyrophil reticulum ramifies throughout the growth, and appears to be derived from the mesenchyme of the membrane.

Normally the epithelial surface of the chorioallantois consists of low cuboidal cells, and is only one cell thick. Under the influence of the Rous and Duran-Reynals tumours it proliferates rapidly and becomes many cells deep (Fig. 5). The cells in the deepest layer are polyhedral, basophilic, and contain large vesicular nuclei, with a prominent nucleolus. They tend to grow down as finger-like processes into the mesenchyme, and even exhibit infiltrative properties (Fig. 6). The more superficial cells are eosinophilic, and either become flattened or grossly enlarged to form a spherical cell exhibiting distinct cell walls surrounding a fine granular eosinophilic cytoplasm and a pale staining vesicular nucleus. Mitotic figures are numerous in all these cell types, and vesicular inclusions are occasionally seen (Fig. 7). The most superficial layer consists of flattened keratinized cells, and large swollen vacuolated cells with pyknotic nuclei. Such cells frequently contain small brilliantly eosinophilic granular aggregations, the nature of which is obscure.

Many of the ectodermal down-growths appear in section as typical " epithelial pearls," or " cell nests." They have an outer layer of basophilic low-cuboidal epithelium corresponding to the deepest layer of the mass of proliferating ectodermal cells of the chorioallantois. Mitotic figures are frequent. Passing towards the centre of the " cell nest," the cells become progressively flattened and keratinized, and the central region is occupied by a structureless, intensely eosinophilic mass of hyaline material.

### (b) *Action of Dermatrophic Virus on the Chorioallantoic Membrane.*

In order to have a basis of comparison for the ectodermal lesions produced by the Rous and Duran-Reynals virus, eggs were inoculated with fowl pox virus, known to produce lesions and typical inclusion bodies in the ectoderm (Woodruff and Goodpasture, 1931), and also with the virus of contagious papilloma (" angle-berries ") of cattle.

*Experiment* 6.—Fowl pox virus was prepared as follows: 0·5 g. of dried vaccine was placed in a sterile tube containing glass beads and 10 ml. normal saline, and the tube was closed with a boiled rubber bung. The tube was shaken in an automatic shaking machine for 1 hour, at the end of which the suspension was filtered through a " Technico " filter, using a Ford's " Sterimat " Grade G.S. The filtrate was used as the inoculum, the amount inoculated on to each membrane varying between 0·02 to 0·06 ml.

Four 10-day embryonated eggs were inoculated, using a graduated capillary pipette as a dropper. In order to secure a wide distribution of the virus over the membrane, it was placed direct on to the shell membrane, and a tear made through this with a glass needle. The virus was then drawn into the egg and on to the chorioallantois by applying negative pressure to the air-cell with the aid of a small rubber teat. The egg was then sealed in the normal way and incubated for 3 days. When opened and examined, small roughly circular opacities were found on the chorioallantois of 3 embryos. The fourth was found to be dead and infected, probably due to accidental cracking of the shell during drilling.

*Histology of lesion.*

The mesenchymal tissue is thickened due to connective-tissue proliferation, oedema, and a fairly intense infiltration of granulocytes. Ectodermal proliferation is marked. The cells are large with vesicular nuclei, and are spherical or polygonal, with a well-defined outline. Definite eosinophilic virus inclusion bodies occur in the cytoplasm (Bollinger bodies) (Fig. 8).

Within the mesenchyme of the chorioallantois islands of epithelial cells occur, also showing inclusions. An occasional attempt at the formation of a " cell nest " may be found, but usually the cells occur in groups without any sign of a whorled appearance. The endothelial aspect of the chorioallantois is much folded in these regions of thickening, but there is no sign of endothelial proliferation.

*Experiment* 7.—Fresh papillomata from a natural case in a bovine were ground up with sand and saline, and the suspension filtered as before to obtain the virus. This was inoculated in the usual way on to the chorioallantois of one 8-day embryo using a dose of 0·1 ml. The egg was opened on the 18th day of incubation and the membrane cut out and examined in saline. No opacities were seen, but the membrane appeared to be a little less flexuous than normal.

Histologically the membrane was thickened, due to proliferation of the mesenchymal tissue. Minute ectodermal proliferations were present, but no definite inclusion bodies were detected, neither were any " cell nests " present.

### (c) *Chemically Induced Tumours* (*Virus free ?*)

Four experiments were performed using chemically induced tumour tissue from the mouse. Two of these experiments were yolk-sac cultivation attempts, the tumours used being methylcholanthrene induced transplantable mammary carcinoma, and the first attempt (Experiment 8) was a complete failure, owing to necrotic changes in the original tumour.

Experiment 9 was a repeat of the above, and of the 13 eggs injected on the 4th day of incubation, all embryos died at various stages save one, which when examined on the 18th day of incubation showed a ring of white tumour tissue surrounding the yolk-sac umbilicus (Fig. 9). The histology of this growth was very similar to that of the original tumour, i.e. carcinoma simplex, with large round and spindle-shaped cells.

Experiments 10 and 11 were chorioallantoic implants using methylcholanthrene induced transplantable mammary carcinomas of mice. Five 10-day embryonated eggs were implanted with fragments of tumour and examined 7 days later. All but one contained small tumours 3–5 mm. diameter. The membranes adjacent to these growths were thickened and somewhat opaque. A definite zone of opacity surrounded all the tumours.

Histological examination of the tumours shows typical carcinoma simplex, but with a good deal of connective-tissue stroma and some areas of necrosis. The surrounding chorioallantois is thickened, mainly due to oedema, and the epithelium shows advanced proliferative changes as previously described, with abundant formation of "cell nests." The epithelial cells of the outermost layer are swollen and cystic, being devoid of cytoplasm and nucleus and leaving only a keratinized cell-border (Fig. 10).

Experiments 12 and 13 with rat tumours (Walker rat carcinoma) resulted in 100 per cent mortality in the 20 embryos receiving implants. Subsequent histological examination showed that the original tumours contained large areas of necrosis.

*Experiment* 14.—Two 10-day embryonated eggs were implanted with a chemically (1:2:5:6-dibenzanthracene) induced fibro-sarcoma of the fowl. The tumour, one of a series so produced by Dr. Peacock of the Glasgow Royal Cancer Hospital, is known as G.R.C.H./15. All attempts to demonstrate a virus in this sarcoma have up to the present been unsuccessful (Peacock, 1946).

The eggs were opened on the 9th and 10th days, and both contained well defined spherical tumours of 5 mm. and 8 mm. diameters respectively. There was no sign of reaction in the membranes surrounding the tumours (Fig. 11).

Histologically G.R.C.H./15 is a dense, vascular fibro-sarcoma, exhibiting many mitotic figures. Cultivated on the membrane the tumour is essentially similar to the original. Some areas are more loosely cellular (Fig. 12) and there is a very rich vascularity, also areas of haemorrhage and necrosis. The ectoderm of the chorioallantois adjacent to the tumour is practically normal. That investing the tumour, however, exhibits proliferation and swollen empty keratinized cells. "Cell nests" are absent from this region, but attempts at their formation may be seen in the body of the tumour by down-growth in the usual way, and also by proliferation of displaced and engulfed epithelial cells by the rapidly growing tumour implants.

(d) *Spontaneous Mouse Tumours.*

*Experiments* 15 *and* 16.—Twenty-eight eggs incubated between 4 to 5 days had injected into the yolk-sac 0·25 ml. of a suspension of mouse sarcoma S.37 in normal saline. Twenty-seven embryos subsequently died at periods varying between 1 and 8 days. Most of these showed congestion and petechial haemorrhages in the skin. No bacteria were isolated from these eggs. One had a small tumour growing in the yolk-sac, attached near the umbilicus. The one surviving chick was removed from the egg on the 19th day, and showed small tumours in the yolk-sac.

S.37 mouse sarcoma is a polymorphous cell sarcoma. The two tumour-bearing eggs showed some diversity of structure in the growths. There were round cell areas, spindle areas, and areas showing both these types of cell. Unfortunately it was not possible to repeat these experiments using the chorioallantoic technique owing to the failure to obtain more S.37 mice. There were six saline injected eggs to serve as controls in these experiments, and they all hatched normally.

*Experiment* 17.—0·25 ml. of a suspension of a transplantable mouse lymphosarcoma were injected into the yolk-sacs of each of nineteen 4–5-day embryonated eggs. All the embryos died at varying periods up to the 11th day following the injection. They were bacteriologically sterile. The dead embryos showed intense congestion and petechial haemorrhages. In 2 cases tumours were found attached to the interior of the yolk-sac. Tumour-bearing membranes were ground up with sand and saline, and 0·25 ml. of the resultant emulsion injected subcutaneously into 10 mice. A haemorrhagic membrane was treated in the same way and injected into 5 mice. Nine days later the mice were found dead, due to a heating failure during a cold spell. Six of the mice injected with tumour-bearing membranes were found to have small lymphoid tumours growing in the subcutis at the site of injection. As with the S.37 experiments, it was not possible to repeat these observations using the chorioallantoic method of cultivation, because no more tumour-bearing mice were available.

*Experiment* 18.—Three 8-day embryonated eggs received implants of fragments obtained at biopsy of an osteogenic sarcoma from the hind leg of a dog. Control nutient broths inoculated with material from this tumour remained sterile.

Nine days later the eggs were opened and no trace of the implants could be found. The membranes appeared normal upon histological examination.

(e) *Spontaneous Chicken Tumours.*

In Experiments 19, 20 and 21 the intra-vitelline method was again tried. These experiments may be briefly dismissed, as in each case contamination with bacteria killed all the eggs. The material for Experiment 19 was a primary liver carcinoma of a chicken. This was injected in the usual manner into 24 4-day embryonated eggs, all of which died within 6 days. *Bact. coli* was isolated from the eggs, although broths inoculated with tumour tissue from the original case remained sterile.

Experiments 20 and 21 consisted of the injection of a saline suspension prepared from lymphocytomata, involving the thigh and ovary respectively. All the embryos died subsequent to injection, and *Bact. coli* and a streptococcus were isolated from the eggs. Material from the thigh lymphocytoma was also injected intramuscularly into a Brown Leghorn chicken with negative results when the bird was destroyed 13 months later.

The next ten experiments consisted of chorioallantoic implants.

*Experiment* 22.—Two 6 to 7-day embryonated eggs were implanted with fragments of an ovarian adeno-carcinoma from a Brown Leghorn hen which had died a few hours previously. Both embryos died within 3 days, and the original material and the eggs were found to be infected with *Bact. coli*.

*Experiment* 23.—Six 7-day embryonated eggs received implants from an ovarian adeno-carcinoma from a Brown Leghorn hen. This tumour had spread in the abdominal cavity by transcoelomic implantation to the mesentery and serosa of the gut and oviduct. Tumour fragments were obtained from the two latter sites. The eggs were opened on the 18th day of incubation, and only one bore a small tumour (3 $\times$ 2 mm.) on the membrane. This was not much bigger than the original implant. Histologically this proved to be necrotic. There was no reaction in the membrane adjacent to the implant.

*Experiment* 24.—Ten 9-day embryonated eggs were implanted with fragments of an ovarian adeno-carcinoma from a Black Leghorn hen. Broths subsequently inoculated with material from this tumour showed contamination with *Bact. coli*. Three embryos died within 2 days of implantation. Of the remainder, 6 developed tumours. These were spherical, yellowish in colour, and measuring about 5 mm. in diameter. An opaque zone of reaction was present in the membranes surrounding the tumours.

Histologically the tumour implants showed a great deal of necrosis. The plain muscle and fibrous tissue moiety had persisted longer than the epithelial elements. The adjacent membrane showed epithelial proliferation, with irregularly shaped small eosinophilic inclusions in the swollen cells. There were no " cell nests." The mesenchyme of the membrane was heavily infiltrated with granular leucocytes.

One of these tumours was re-implanted on to the chorioallantois of a 9-day embryo, with the results given below.

*Experiment* 25.—This died on the 5th day, due to streptococcal infection. The graft was found to be necrotic, and there was no membrane reaction in its vicinity.

*Experiment* 26.—Fragments of a friable ovarian adeno-carcinoma from a cross-bred hen were implanted on the membranes of four 10-day embryonated eggs. Broths inoculated at the same time with tumour tissue showed a streptococcus in pure culture on the following day. Examination of the eggs by trans-illumination, however, showed the embryos to be alive. They were examined on the eighth day, and three were found to contain tumours on the chorioallantois. These varied between 2 and 3·5 mm. in diameter. There were no detectable lesions on the membrane.

The original growth had the appearance of a granulosa-cell tumour exhibiting a " cylindroid " structure. On the membrane this is not so apparent, mainly owing to the growth of the epithelial cells at the expense of the tumour trabeculae of connective tissue, and to a gross infiltration of eosinophilic granular leucocytes. There is some proliferation of the ectodermal cells of the membrane and a few " cell nests " are present.

*Experiment* 27.—Two of the tumours grown on the chorioallantois in the previous experiment were re-implanted into two 8-day embryonated eggs. These were opened 10 days later and both contained small yellowish growths on the membrane, surrounded by many minute irregular opacities extending for 7–8 mm. each side of the implant.

Histological examination of one membrane shows a few islands of surviving tumour cells, general thickening, ectodermal proliferation, and the presence of large numbers of well-formed " cell nests."

The remaining membrane was ground with sterile sand and saline, and the resulting suspension injected intraperitoneally into two 6-week-old Brown Leghorn chickens (females) with negative results when the birds were destroyed 18 months later.

*Experiment* 28.—Three 9-day-old embryos received implants on to the membranes with fragments of both liver and spleen from a R.I.R. hen suffering from aleukaemic lymphoid leucosis. Eight days later two of the embryos were found to be dead, namely those with liver and liver plus spleen implants. Only the latter had a tumour in the membrane. The remaining spleen only implanted embryo was living, and the membrane bore a 4 mm. diameter pink tumour. In the case of the dead embryos both were extremely congested, and the livers appeared swollen and leukaemic.

Histological examination of the membrane bearing the spleen implant shows a vascular growth of endothelial-like structure. All resemblance to normal or even leukaemic spleen has disappeared. The growth is composed of an irregular syncytium of cells with round, angular or elongated nuclei, the cytoplasm of which is drawn out into processes which appear to merge with those of adjacent cells. Rounded deeply staining cells (haemocytoblasts ?) lie in the interstices of this syncytial stroma. The vessels are formed of a single layer of flattened endothelium and are congested. At the periphery of this tumour large numbers of primitive blood cells appear to be wandering into the mesenchymal stroma of the chorioallantois. " Cell nests " occur in this region. One or two small areas of necrosis in the centre of the tumour are surrounded by giant cells containing many nuclei. " Cell nests " arising from ectodermal proliferation are present in the membrane adjacent to the implant.

The liver plus spleen implant shows an essentially similar picture to the above, as the liver moiety has disappeared. In its place is a dense scar of connective tissue and collagen, infiltrated with various types of wandering cells. The adjacent membrane shows proliferation of ectoderm and the formation of " cell nests."

The livers from the two dead embryos show a typical leukaemic picture (Fig. 13). The infiltrating round cells are primitive, and can only be designated by the term haemocytoblasts. An examination of the heart blood from these two embryos shows in the case of the liver only implant a large number of primitive cells which appear to be differentiating into erythroblasts, i.e. erythroleucosis, and in the second case (liver + spleen implant) a typical myeloid leukaemia (Fig. 14). Blood and tissues from the living (spleen only implant) embryo show no deviation from the normal.

*Experiment* 29.—Three 5-day embryonated eggs received 0·05 ml. blood from the living embryo which had the leukaemic spleen implanted on the chorioallantois. The blood was simply dropped on to the exposed membranes. One embryo was examined on the 17th day and found to be normal, as also was the membrane. The other two hatched out normally on the 21st day, and remained normal until they were killed 18 months later.

*Experiment* 30.—Four 3-day embryonated eggs were implanted on the chorioallantois with fragments of tumour tissue from a case of multiple lymphocytoma of the liver of a R.I.R. hen. The bird was destroyed and immediately opened, and the viscera were exposed to the air in the post-mortem room for about 2 hours before the case was seen, but the liver surface was seared with a hot spatula at the time of implantation, and fragments taken from within the organ with sterile knife and forceps. The actual implants were made about 2½ hours after the fowl was destroyed. Broths inoculated with tumour material at the same time were later seen to be sterile.

Nine days later two embryos were found to be dead. They were congested and a blood examination showed large numbers of leucocytes, which were degenerated as the result of autolysis, which proceeds rapidly at incubator temperature. The third chick hatched normally and survived for two days. Blood films and sections of liver and spleen from this case were normal.

*Experiment* 31.—Six 10-day embryonated eggs were implanted with fragments of a teratoma arising in the right kidney of a Light Sussex hen. The tumour weighed 580 g. (Fig. 15).

Four days later three of the embryos were found to be dead and congested. They were bacteriologically sterile, as was the original tumour. Of the remaining eggs, two contained tumours 5 mm. diameter, and well supplied with blood vessels from chorioallantois. The growths were surrounded by an intense haemorrhagic zone (Fig. 16). The other egg contained an apparently normal embryo, and the membrane was free from tumour implant.

Histologically the original tumour was composed of embryonic renal tubules reminiscent of the developing metanephros, a connective-tissue moiety with a sarcomatous appearance, plain muscle fibres, circular islands of cartilage, and tubules composed of ciliated mucous secretory cells suggesting tracheal or bronchial epithelium (Fig. 17). There were large areas of haemorrhage and necrosis.

The two cultivated tumours (4 and 7 days respectively) both show a vascular sarcomatous-looking connective-tissue stroma, in which are embedded a few scattered tubules of epithelial cells, resembling those of embryonic kidney. Numerous bundles of plain muscle fibres are scattered throughout the tumour. There is a distinct ectodermal proliferation of the membrane adjacent to the tumour, and many " cell nests " are present in this region, as well as in the mesenchyme investing the growth (Fig. 18).

The results of the preceding experiments are summarized in Table I. It will be seen that the reaction of the chorioallantois has been somewhat arbitrarily divided into simple ectodermal proliferation and " cell nest " formation. These reactions of the membrane ectoderm differ only in degree; since simple proliferation may or may not be followed by " cell nest " formation, according to the nature of the stimulus.

### DISCUSSION.

It is not necessary to emphasize the importance of an easy method of cultivation of tumours. Until the egg cultivation techniques were applied to cancer research tumours could only be propagated and studied in the laboratory either by direct homologous implantations or tissue culture. The first method is not

TABLE I.

| Implant. | Virus. | Exp. No. | Reaction of adjacent membrane: Ectodermal proliferation. | Reaction of adjacent membrane: "Cell nest" formation. |
|---|---|---|---|---|
| Rous I sarcoma | + | 1 | + | + |
| | | 2 | + | + |
| | | 3 (infected) | — | — |
| | | 4 (virus only) | + | + |
| Duran-Reynals sarcoma | + | 5 | + | + |
| — | Fowl-pox | 6 | + | + |
| — | Contagious papilloma (bovine) | 7 | + | — |
| Chemically induced mouse mammary carcinoma | — ? | 10 | + | + |
| | | 11 | + | + |
| GRCH/15 | — ? | 14 | + | + |
| Osteogenic sarcoma (dog) | ? | 18 (failed to grow) | — | — |
| Spontaneous adeno-carcinoma (chicken) | ? | 22 (infected) | — | — |
| | | 23 (failed to grow) | — | — |
| | | 24 (infected) | + | — |
| | | 25 " | — | — |
| | | 26 | + | + |
| | | 27 | + | + |
| Lymphoid leucosis (chicken) | + | 28 | + | + |
| | | 30 (embryos died) | Membranes unsuitable for examination. | |
| Embryonal nephroma (chicken) | ? | 31 | + | + |
| | | CONTROLS. | | |
| Normal adult chicken :— | | | | |
| Liver | — | — (failed to grow) | — | — |
| Spleen | — | — (infected) | + (also endoderm) | + |
| Liver | — | — | — | — |
| Kidney | — | — | — | — |
| Ovary | — | — | — | — |
| Testis | — | — | — | — |
| Spleen | — | — | — | — |
| Chick embryo :— | | | | |
| Gut | — | — | + | + |
| Liver | — | — | + | + |

dependable, since in many cases it has been observed that tumours will only grow successfully in hosts closely related to the donor, a condition not always easy to fulfil, especially with unusual tumours growing in unusual hosts. The second method of tissue culture is involved and requires an elaborate technique, and has the great drawback that frequently only one component of the original neoplasm grows, while the rest, e.g. the stroma, tend to disappear.

When the yolk-sac technique was described in 1942 by Taylor *et al.* it seemed from their paper that a very easy and reliable method of cultivation of heterologous neoplastic tissue was at last available to workers in the cancer field. Other workers (Hungate *et al.*, 1944 ; Heilman and Bittner, 1944 ; Bittner *et al.*, 1945) also tried this method, and claimed good results with the particular tumours with which they worked—mainly derived from the rat and mouse.

The method, however, was not found so reliable by Twombly and Meisel (1946), who reported a very high mortality in their embryos, a disappointingly small percentage of successful takes, and complete failures with several types of tumour.

These findings have been confirmed by the present investigation, where, in a number of experiments, in which 157 eggs were injected with various mouse, rat and chicken tumours, both spontaneous and chemically induced, growths developed in the yolk-sac in only five instances.

It should not be thought, however, that the method is therefore of little value. In some hands it appears reasonably reliable, whilst in others not so reliable, and certainly wasteful of fertile eggs.

Certain results obtained with this yolk-sac inoculation technique are interesting and are being further investigated. For instance, in a number of cases embryos developed haemorrhages and died at varying periods subsequent to injection of either tumour suspension or tumour filtrate. These deaths were not always due to bacterial contamination, and could tentatively be ascribed to some lethal agent such as a virus, though the possibility that death was caused by the introduction of foreign proteins into the food supply of the embryo must not be overlooked. More experiments involving sub-inoculation with tissue filtrates and extra-embryonic fluids from dead and haemorrhagic embryos should help to settle this point.

The chorioallantoic method has been found to be most successful in the case of the cultivation of avian tumours associated with viruses. Thus, the Rous I sarcoma and the Duran-Reynals sarcoma give the biggest growths in the membrane.

These are both rapidly growing tumours, and therefore would naturally tend to give better results in the short period (never more than 14 days) of cultivation. Slow growing tumours usually give disappointingly small growths, whilst normal adult tissues may fail to grow and be absorbed.

In the case of the Rous and Duran-Reynals sarcomata, the virus stimulates the mesenchymal tissue of the chorioallantois to participate in the general malignancy, thus augmenting the size of the implant. Of more interest, however, is the observation, originally by Keogh (1938), that the Rous virus also appears to stimulate the ectodermal cells of the chorioallantois to proliferate in an apparently uncontrolled manner. He speculates on the possibility that the virus may be carcinogenic, not only for mesenchymal tissue, but also for epithelium under certain conditions. Ectodermal proliferation due to Rous virus has been confirmed in the present investigation, and the pathological changes in the membrane ectoderms produced by the Rous and Duran-Reynals tumours have been compared with those produced by dermatrophic viruses such as those of fowl-pox and contagious papillomata of cattle, also with similar changes noted in the membrane adjacent to certain implanted spontaneous tumours of the chicken. As a check on these observations a number of experiments were performed with chemically induced tumours of the mouse, rat and chicken ; also with normal adult and embryonic chicken tissues.

The significance of the " cell nests " or " epithelial pearls " is interesting, resembling as they do transverse sections of infiltration cores of epithelial tissue which characterize squamous-cell carcinoma. Similar lesions were noted by Huxley and Murray (1924) adjacent to the fragments of chick embryos implanted on the chorioallantois.

It is well known that embryonic tissue extracts contain growth-promoting substances, and for this reason are frequently added to tissue culture media. Claude (1938) showed that a fraction could be isolated from chick embryos which possessed the same physical and chemical properties as the purified active fraction of the Rous I sarcoma. Despite these similarities, however, the embryo extract failed to produce tumours in susceptible chickens.

In this connection it is interesting to note that Earle (1943) noticed an apparent malignant transformation of mouse fibroblasts grown on a fibrin clot and bathed in horse serum and chick embryo juice.

These observations, together with the production of ectodermal proliferation and cell nest formation found to be associated with so many chorioallantoic implants of tumours in the present investigation, gave rise to the conception that tumours and embryonic tissue might contain growth-promoting substances capable of causing the overgrowth of the ectodermal layer of cells in the embryonic membrane.

If this proved to be the case it might be possible to demonstrate some relationship between the intensity of the membrane reaction and the type of tumour implanted, especially from the aspect of virus content. No mention of " epithelial pearls " is made by Keogh (1938), or by the numerous other workers who have studied ectodermal lesions of the chorioallantoic membrane induced by viruses.

A number of experiments were devised in order to test this hypothesis, and especially to study the membrane reaction to a spontaneous liver carcinoma of the duck, which is described in a separate paper.

Table I shows that the ectodermal lesions do not appear to be as specific as was hoped. In all cases where tumours known to contain virus, or filtrates of such tumours, or dermatrophic viruses, were placed on the membrane, proliferation of the ectoderm took place. In some instances eosinophilic " inclusions " were observed, but the nature of these is not clear, except of course in the case of fowl pox. In the main, virus alone caused simple proliferation without the formation of " cell nests."

In general those tumours which failed to grow subsequent to implantation and were absorbed did not cause any detectable lesions in the membrane ; similar results were obtained for normal adult tissues. Also in several cases, although not invariably, an accidental infection of the membrane with bacteria did not result in ectodermal proliferation.

The spontaneous chicken tumours which grew successfully all caused proliferation and the formation of " cell nests."

It was thought possible that chemically induced tumours, being presumably free of virus, might give a different membrane reaction. The G.R.C.H./15 (1:2:5:6-dibenzanthracene) induced fowl sarcoma did not give rise to lesions in the membrane adjacent to the growth, but there were proliferative changes in the ectoderm investing the tumour, and atypical " cell nests " were seen in the interior of the growth. The two chemically-induced mammary tumours of mice, however, both gave rise to typical reactions in the adjacent membranes.

The induction of leukaemic myeloid leucosis in one embryo, and erythroleucosis in a second embryo following implantation of liver and spleen from a case of leukaemic lymphoid leucosis, and of an undiagnosed leukaemia in an embryo following the implantation of lymphocytoma of the liver are of interest, and appear to confirm the findings of Pierce (1942). Uhl, Engelbreth-Holm and Rothe Meyer (1936) report that the virus from a case of stem-cell leukaemia in the chicken may induce myeloid leucosis, erythroleucosis or even sarcomata according to the developmental stage of the cell (haemopoietic or a near relative, either ancestral or descendant) which is attacked.

### SUMMARY AND CONCLUSIONS.

Various *in vivo* methods of cultivation of spontaneous and chemically induced avian and mammalian tumours have been tried, and the best results have been obtained with the chick embryo chorioallantoic membrane method.

Virus-associated tumours grow best, followed by the chemically induced tumours. The majority of spontaneous tumours give disappointingly small growths.

It has been shown that the capacity for inducing ectodermal proliferation in the chorioallantois is not confined to tumour viruses, or to fowl pox and other non-tumour associated viruses, but occurs in the membranes adjacent to the majority of implanted tumours, whether spontaneous, virus associated, or chemically induced; or even occasionally in response to bacterial growth. Attention has been drawn to the tendency of the proliferating ectoderm to form " cell nests " or " epithelial pearls," resembling those occurring in squamous-cell carcinoma, and the significance of this has been discussed.

The induction has been described of two distinct leukaemic conditions in chick embryos as the result of implanting tissues from a third type of spontaneous leukaemia in a fowl, and has been briefly discussed.

I wish to express my gratitude to Professor A. Murray Drennan, of the Pathology Department, University of Edinburgh, for his interest in this work, and for the numerous suggestions as to the manner of development of the investigation.

All the fertile eggs were obtained from Dr. A. W. Greenwood's flock of Brown Leghorns, and I have pleasure in recording my appreciation of his co-operation in this respect, and for much help in other directions.

The virus associated and chemically induced tumours were supplied by Dr. J. G. Carr, formerly of the Institute of Animal Genetics, University of Edinburgh, to whom I am much indebted.

The histological preparations were the work of Messrs. Cockburn and MacKenzie.

Part of the expenses incurred by this work were borne by the British Empire Cancer Campaign.

### REFERENCES.

BEVERIDGE, W. I. B., AND BURNET, F. M.—(1946) *Spec. Rep. Ser. med. Res. Coun., Lond.*, No. 256.

BITTNER, J. J., EVANS, C. A., AND GREEN, R. G.—(1945) *Science*, **101**, 95.

CAMPBELL, J. G.—(1945) *J. comp. Path.*, **55**, 308.

CARR, J. G.—(1943) *Brit. J. exp. Path.*, **24**, 127.

CLAUDE, A.—(1938) *Proc. Soc. exp. Biol., N.Y.*, **39**, 398.

EARLE, W. R.—(1943) *J. nat. Cancer Inst.*, **4**, 165.

GRASSETT, E. C.—(1929) *Publ. S. Afr. Inst. med. Res.*, No. 24.

HEILMAN, F. R., AND BITTNER, J. J.—(1944) *Cancer Res.*, **4**, 578.

HUNGATE, R. E., SNIDER, H., TAYLOR, A., AND THOMPSON, R. C.—(1945) *Univ. Tex. Publ.* No. 4507, " Cancer Studies," 75.

*Idem*, TAYLOR, A., AND THOMPSON, R. C.—(1944) *Cancer Res.*, **4**, 289.

HUXLEY, J. S., AND MURRAY, P. D. F.—(1924) *Anat. Rec.*, **28**, 385.

KEOGH, E. V.—(1938) *Brit. J. exp. Path.*, **19**, 1.

MINOURA, T.—(1921) *J. exp. Zool.*, **33**, 1.

MURPHY, J. B.—(1914) *J. exp. Med.*, **19**, 181.

PEACOCK, P. R.—(1946) *Cancer Res.*, **6**, 311.

PIERCE, M.—(1942) *Amer. J. Path.*, **18**, 1127.

POLK, A., BUDDINGH, G. J., AND GOODPASTURE, E. W.—(1938) *Ibid.*, **14**, 71.

ROUS, P., AND MURPHY, J. B.—(1911) *J. Amer. med. Ass.*, **56**, 741.

STEVENSON, H. N.—(1918) *J. Cancer Res.*, **3**, 63.

TAYLOR, A.—(1943) *Science*, **97**, 123.

*Idem*, THACKER, J., AND PENNINGTON, D.—(1942) *Ibid.*, **96**, 342.

TWOMBLY, C. H., AND MEISEL, D.—(1946) *Cancer Res.*, **6**, 82.

UHL, E., ENGELBRETH-HOLM, J., AND ROTHE MEYER, A.—(1936) *Acta path. microbiol. scand.*, **13**, 394.

WOODRUFF, A. M., AND GOODPASTURE, E. W.—(1931) *Amer. J. Path.*, **7**, 209.

### INTRANASAL ADMINISTRATION OF MAMMARY TUMOUR MILK FACTOR.

A. M. BEGG.